# Digital Food Frequency Questionnaire Assessing Adherence to the Norwegian Food–Based Dietary Guidelines and Other National Lifestyle Recommendations: Instrument Validation Study

**DOI:** 10.2196/53442

**Published:** 2024-04-30

**Authors:** Hege Berg Henriksen, Markus Dines Knudsen, Anette Hjartåker, Rune Blomhoff, Monica Hauger Carlsen

**Affiliations:** 1 Department of Nutrition Institute of Basic Medical Sciences University of Oslo Oslo Norway; 2 Section for Colorectal Cancer Screening Cancer Registry of Norway Oslo Norway; 3 Department of Transplantation Medicine Division of Surgery, Inflammatory Diseases and Transplantation Oslo University Hospital Oslo Norway; 4 Department of Clinical Service Division of Cancer Medicine Oslo University Hospital Oslo Norway

**Keywords:** digital food frequency questionnaire, lifestyle assessment tool, relative validity, physical activity, Norwegian food–based dietary guidelines, Norway, Norwegian, food, foods, diet, dietary, lifestyle, assessment, digital questionnaire, investigation, food intake, dietary intake, dietary intakes, observation, monitoring, youths, adolescent, adolescents, teen, teens, teenager, teenagers, cross-sectional study

## Abstract

**Background:**

Valid assessment tools are needed when investigating adherence to national dietary and lifestyle guidelines.

**Objective:**

The relative validity of the new digital food frequency questionnaire, the DIGIKOST-FFQ, against 7-day weighed food records and activity sensors was investigated.

**Methods:**

In total, 77 participants were included in the validation study and completed the DIGIKOST-FFQ and the weighed food record, and of these, 56 (73%) also used the activity sensors. The DIGIKOST-FFQ estimates the intake of foods according to the Norwegian food–based dietary guidelines (FBDGs) in addition to lifestyle factors.

**Results:**

At the group level, the DIGIKOST-FFQ showed good validity in estimating intakes according to the Norwegian FBDG. The median differences were small and well below portion sizes for all foods except “water” (median difference 230 g/day). The DIGIKOST-FFQ was able to rank individual intakes for all foods (*r*=0.2-0.7). However, ranking estimates of vegetable intakes should be interpreted with caution. Between 69% and 88% of the participants were classified into the same or adjacent quartile for foods and between 71% and 82% for different activity intensities. The Bland-Altman plots showed acceptable agreements between DIGIKOST-FFQ and the reference methods. The absolute amount of time in “moderate to vigorous intensity” was underestimated with the DIGIKOST-FFQ. However, estimated time in “moderate to vigorous intensity,” “vigorous intensity,” and “sedentary time” showed acceptable correlations and good agreement between the methods. The DIGIKOST-FFQ was able to identify adherence to the Norwegian FBDG and physical activity recommendations.

**Conclusions:**

The DIGIKOST-FFQ gave valid estimates of dietary intakes and was able to identify individuals with different degrees of adherence to the Norwegian FBDG and physical activity recommendations. Moderate physical activity was underreported, water was overreported, and vegetables showed poor correlation, which are important to consider when interpreting the data. Good agreement was observed between the methods in estimating dietary intakes and time in “moderate to vigorous physical activity,” “sedentary time,” and “sleep.”

## Introduction

Monitoring dietary intake and physical activity with valid and feasible methods in a population is of great importance, as there is a growing body of evidence on the strong association between lifestyle and risk of several chronic diseases [1-5].

Even though self-administered web-based 24-hour dietary recalls and food are increasingly used in epidemiological studies [6,7], the most common type of dietary assessment tool in observational and intervention studies is still the food frequency questionnaire (FFQ) [8,9]. Generally, the FFQs assess the frequency of food intakes and portion size of the food consumed, are typically self-administrated, and vary with regard to which food items or nutrients are included, portion sizes, frequency categories, length of the questionnaire, and duration of the registration period (ie, weeks, months, or years) [6,10-12]. The FFQs may be used to estimate habitual dietary intakes over a longer period of time and to rank individuals according to dietary intakes and may generate data on dietary intakes on a group level. Thus, FFQs are valuable in assessing the degree of adherence to dietary recommendations in a population [8,9,13-15].

As with all dietary assessment methods, FFQs are prone to reporting errors, particularly systematic errors, often related to memory, knowledge of portion sizes, and understanding of the questions [11,12,16,17]. This can result in over- or underestimation of dietary intakes [18,19], which may attenuate the observed association between diet and health outcomes [20,21]. Systematic error may be reduced with clear instruction and the use of relevant examples, such as pictures of portion sizes and informative text.

The emerging use of digital applications has resulted in modifications of traditional printed assessment tools into web-based tools in both research and the clinic [22,23]. Web-based dietary assessment tools have demonstrated good validity and give comparable data with printed versions [6,23-25].

We have developed a short digital semiquantitative food and lifestyle frequency questionnaire (the DIGIKOST-FFQ) based on a validated printed questionnaire called NORDIET-FFQ [26] developed at the Department of Nutrition, University of Oslo, Norway. The DIGIKOST-FFQ is designed to assess adherence to the Norwegian food–based dietary guidelines (FBDGs) and national lifestyle guidelines [3,27] and has been revised according to the results from a qualitative evaluation study (ie, focus group interviews and usability testing) [27].

Generally, the DIGIKOST-FFQ delivers similar data on the main dietary and physical activity recommendations as the NORDIET-FFQ. However, some adjustments have been made based on the results from the validation study of the NORDIET-FFQ (Multimedia Appendix 1 [3,26-38]). In addition, the DIGIKOST-FFQ is based on a digital platform constituting several technical functions to improve the feasibility and the understandability of the questions [27].

Therefore, investigating the relative validity of this new digital assessment tool is of great importance to current and future studies applying the assessment method for dietary data collection. Thus, in this project, we aimed to investigate the relative validity of the DIGIKOST-FFQ in assessing dietary intake, physical activity, and adherence to the Norwegian FBDG in an adult and healthy population in Norway, with weighed food record (WR) and activity sensor as the reference methods.

## Methods

### Study Design and Participants

Participants were recruited between April and September 2021 and randomly extracted by the National Registry of Norway and by advertisements on Facebook. Adult (18 years and older) men and women living in the Oslo region in Norway were eligible for the study. The study design was cross-sectional. All participants completed the DIGIKOST-FFQ, and after 1-2 months, they filled in a 7-day WR and used an activity sensor (SenseWear Armband Mini [SWA]; BodyMedia). In addition to the WR, the participants received a digital scale and an instruction on how to weigh and record all foods and beverages consumed during a period of 7 consecutive days. Due to the COVID-19 pandemic, the instructions were given in a video meeting (Zoom; Zoom Video Communications) by the responsible researchers in the study. Then, the participants were invited to meet outside the study center at the Department of Nutrition, University of Oslo at a pickup point to receive the WR, a digital scale, SWA, and the prepaid postal envelopes for returning the equipment after use.

During the WR registration period, the participants also wore the SWA to record all physical activity, sedentary time, and sleep. By the end of the registration period, the participants returned the completed WRs and the SWA to the study center by postal mail. At the end of the study, all participants were offered to voluntarily fill out the DIGIKOST-FFQ once more in order to receive an individual feedback report benchmarking their dietary intakes and physical activity against the Norwegian FBDG. In addition, they could be randomly selected to win a gift certificate of NOK 500 (US $56.91). The digital scale was also given to the participants as a gift.

### DIGIKOST

The first version of the DIGIKOST-FFQ underwent evaluation in focus group interviews and usability testing [27]. The final version of the DIGIKOST-FFQ is described in Multimedia Appendix 1. In brief, the DIGIKOST is a digital diet and lifestyle assessment tool designed to assess dietary intake and other lifestyle factors and evaluate these according to the Norwegian FBDG. DIGIKOST also has a report function, the DIGIKOST report, which is an individual feedback report on respondents’ adherence to the Norwegian FBDG, with specific and personalized advice on how to fulfill the recommendations**.** The DIGIKOST-FFQ is based on a software platform called Nettskjema, developed and administered by the University Information Technology Center at the University of Oslo, Norway [28]. The main login option in DIGIKOST is the ID-port (e-ID used by the Norwegian Agency for Public Management and eGovernment [Difi]) [29,30]. The DIGIKOST-FFQ takes approximately 20 minutes to complete [27] and includes 103 food and lifestyle items, of which 78 questions about food items (grams per day), 7 questions about physical activity (minutes per week), sedentary time, and sleep (hours per day), 8 questions about tobacco use, and 10 questions about body weight and demographic data. The main food groups cover foods rich in fiber (ie, fruits and berries, vegetables, and whole grain products), fish, dairy products, meat, oils, margarine, and beverages (Table S1 in Multimedia Appendix 1). The responses from the DIGIKOST-FFQ are directly transferred into a secure server called services for sensitive data, and the crude variables are automatically transformed by unique algorithms into food groups, activities, and lifestyle indices according to the national recommendations [26]. The DIGIKOST data set also contains 2 indices, the Norwegian diet index and the Norwegian lifestyle index [32] (Multimedia Appendix 1). The Norwegian diet index consists of 12 components corresponding to the Norwegian FBDG with a 3-level scoring approach including 3 categories representing low, intermediate, and high adherence, giving a composite diet index ranging in scores from 0 (lowest adherence) to 20 (highest adherence) points. The Norwegian lifestyle index consists of 5 components (ie, diet, physical activity, normal weight, alcoholic drinks, and tobacco use) with a 3-level equal scoring approach, and a composite lifestyle index ranging from 0 to 5 points.

### Seven-Day WR

Dietary data were retrieved from the WR and manually coded and imported into the food and nutrient calculation system, KBS (version 7.3, 2018, AE-10 database), developed at the Department of Nutrition, University of Oslo [39].

### Physical Activity Sensor

We used the activity sensors called SWA to objectively measure different activity intensities. The SWA was placed on the nondominant arm at the upper part around the triceps muscle. The participants were instructed to use the SWA all day and night and to only remove it during water-based activities like showers or swimming. All activity data generated from the SWA were exported to a computer, and all calculations on activities were conducted in the SenseWear Professional software (version 7.0; BodyMedia Inc).

### Sample Size

An adequate number of participants in validation studies are shown to be 80-100 [10,11,40,41]. This ensured statistical power of 80% to detect differences of 1 portion of “fruit” or “vegetable” per day (1 portion=100 g) between test and reference methods, assuming an SD of 1.6 portions (or 160 g) and a significance level of 5% [42,43]. Moreover, in order to detect a Pearson correlation coefficient of 0.5 or higher between the DIGIKOST-FFQ and the reference methods, a sample size of 38 men and 38 women is required to achieve a significance level of 5% and a power of 90% [44].

### Statistical Analyses

All analyses were performed using SPSS software (version 29; IBM Corp). Normal distribution for continuous variables was checked by visual inspection of histograms and quantile-quantile plots. Dietary and activity variables were presented in median and IQR. Comparisons between the methods were presented as median differences, and categorical variables were presented in frequencies and percentages. Paired 2-tailed *t* tests were used to compare differences in normal distributed variables, and Wilcoxon signed rank test in nonnormal distributed variables. *P* values <.05 were considered statistically significant. Ranking of individual dietary intakes and activity levels were tested by Spearman ρ for nonparametric variables and the 95% CI for the correlation estimate. A correlation below 0.3 was defined as poor, whereas a correlation between 0.3 and 0.49 was fair, and above 0.5 was satisfactory, according to Hankin et al [45].

Cross-classification of individual intakes between the methods was estimated by ranking participants’ intakes, dividing them into quartiles, and calculating how many were classified in the same and same plus adjacent, misclassified with 2 quartiles, or classified in the opposite quartile (grossly misclassified, 3 quartiles apart).

Bland-Altman plots were used to explore and visualize bias, such as under- or overreporting (mean differences), limits of agreement (mean difference and 1.96 SD), and the presence of outliers in the data. Sensitivity and specificity were calculated to evaluate the participants’ adherence to the Norwegian FBDGs identified by both methods. The sensitivity was defined as the proportion of participants who were classified as not fulfilling the recommendations both by the DIGIKOST-FFQ and the WR or SWA divided by the number of participants not fulfilling the recommendations according to the WR or SWA only. Specificity was defined as the proportion of participants who were classified as fulfilling the recommendations both by the DIGIKOST-FFQ and WR or SWA divided by the number of participants fulfilling the recommendations according to the WR or SWA only. Values above 60% were defined as good for both measures. Adherence to the Norwegian diet index and physical activity recommendations were examined on a continuous scale. For all the different diet and physical activity recommendations, participants were assigned points of adherence, defined as high (1 point), intermediate (0.5 point), and low (0 point) to each specific recommendation.

### Ethical Considerations

This study was carried out in accordance with the Helsinki Declaration. The Norwegian Data Protection Services (Sikt, project registration 277679) has approved the DIGIKOST protocol and the informed consent. The participant signed the informed consent electronically prior to participating in the study. They were also informed about the ability to opt out of the study as well as information regarding the safe storage of all personal data. All data were analyzed in the safe and approved server, and with restricted access, called services for sensitive data at the University of Oslo [28]. Only tables and figures without individual sensitive data were exported. No data were collected about the invited participants who did not participate.

## Results

### Participant Characteristics

**Figure 1:**
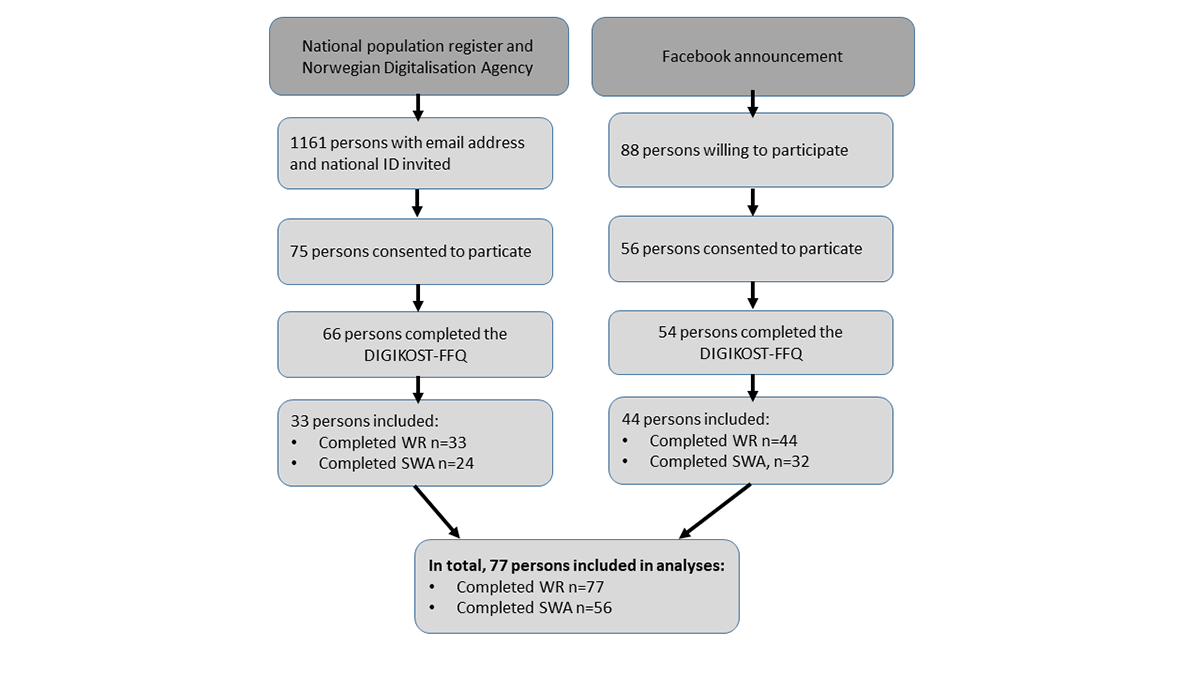
Flow of participants. FFQ: food frequency questionnaire; SWA: SenseWear Armband Mini; WR: weighed food record.

In total, 77 (male: n=16 and female: n=61) participants, corresponding to 6% (n=77) of the 1249 who were invited and 59% (n=77) of the 131 who consented, participated in this study. All of these 77 participants fulfilled the inclusion criterion by the completion of both the DIGIKOST-FFQ and the WR. Of these, 56 (73%) also used the SWA (Figure 1 and Table 1). The mean age was 45 (SD 14.6) years, and the mean BMI was 24.5 (SD 3.9) kg/m^2^. Most of the participants were highly educated (college or higher: n=70, 91%) and currently employed (n=53, 69%). Total daily energy intake estimated from the WR was on mean 8057 (SD 2157) kJ, and energy expenditure measured from the SWA was on mean 10,365 (SD 1827) kJ/day (Table 1).

**Table 1:** Participant’s characteristics, anthropometrics, and demography (N=77).

			Values
**General characteristics**			
	**Gender, n (%)**		
		Men	16 (21)
		Women	61 (79)
	Age (years), mean (SD)		45.2 (14.6)
	Energy intake per day (kJ), mean (SD)		8057 (2157)
	**Days between completion of tools**		
		Mean (SD)	19.4 (16.8)
		Range	1-94
**Activity level per day (n=56), mean (SD)**			
	Total energy expenditure (kJ)		10,365 (1827)
	Active energy expenditure (kJ)		4884 (1680)
	Steps		8868 (3780)
**Anthropometry, mean (SD)**			
	Height (cm)		170.4 (8.1)
	Weight (kg)		71.4 (12.8)
	BMI (kg/m^2^)		24.5 (3.9)
**Education, n (%)**			
	Upper secondary school		7 (9)
	College or university		70 (91)
**Ethnicity, n (%)**			
	Asia		1 (1)
	Europe		74 (96)
	Other country		2 (3)
**Work status, n (%)**			
	In work or employed		53 (69)
	Student		9 (13)
	Retired		6 (8)
	Sick leave and unemployed		8 (10)

### Dietary Intakes From the DIGIKOST-FFQ and WR

#### Overview

Estimates of median dietary intakes from DIGIKOST-FFQ and WR, median differences, correlation coefficients, and cross-classifications are presented in Table 2. All estimated intakes of food groups from the DIGIKOST-FFQ were significantly different from the WR estimated intakes, except for processed meat, sugar-rich beverages, and dietary supplements.

**Table 2:** Estimated intake of food groups from DIGIKOST-FFQ^a^ and WR^b^ for all participants (N=77).^c^

Food group	DIGIKOST-FFQ (g/day), median (P10-P90)	WR (g/day), median (P10-P90)	Difference (g/day), median (P10-P90)	*P*value^d^	Spearman ρ (95% CI)	Correct quartile (%)	Correct+adjacent quartile (%)	Misclassification 2 quartiles (%)	Gross misclassification (%)
Fruits and berries	189 (81-431)	157 (41-495)	31 (–107 to 203)	.02	0.64 (0.48-0.76)	42	87	12	1
Berries	29 (0-93)	14 (0-90)	6.5 (–41 to –86)	.02	0.35 (0.13-0.54)	42	71	21	8
Vegetables	212 (107-414)	150 (53-304)	45 (–120 to 244)	<.001	0.24 (0.0-0.45)	30	69	21	10
Legumes	14.0 (0-57)	0 (0-61)	1.6 (–33 to 37)	.03	0.45 (0.25-0.62)	30	84	16	0
Unsalted nuts	9 (0-30)	0 (0-18)	2.3 (–6 to 26)	<.001	0.48 (0.28-0.64)	50	74	13	13
Whole grains	77 (34-155)	39 (11-84)	43 (–10 to 94)	<.001	0.51 (0.32-0.67)	47	81	17	3
Fish	60 (18-146)	43 (0-120)	22 (–27 to 84)	<.001	0.52 (0.33-0.67)	41	80	17	3
Fatty fish	28 (10-83)	28 (0-79)	10 (–23 to 45)	.002	0.61 (0.44-0.74)	43	82	18	0
Low-fat dairy products	70 (0-351)	150 (1-370)	–37 (–228 to 137)	<.001	0.65 (0.49-0.77)	47	86	12	3
High-fat dairy products	14 (3-131)	31 (6-72)	–9.4 (–51 to 70)	<.001	0.33 (0.11-0.52	35	73	20	8
Red meat	35 (0-105)	54 (0-145)	–13 (–77 to 35)	.002	0.58 (0.40-0.72)	48	84	13	3
Processed meat	29 (0-96)	38 (0-101)	–2.5 (–60 to 40)	.13	0.54 (0.35-0.69)	51	82	13	5
Water	1100 (300-1737)	757 (94-1968)	230 (–737 to 1020)	.01	0.43 (0.22-0.59)	42	79	17	4
Sugar-rich beverages	0.0 (0-69)	28 (0-95)	0.0 (–60 to 57)	.14	0.30 (0.08-0.50)	58	82	18	0
Ethanol	6 (0-20)	8 (0-29)	–0.8 (–17 to 5)	.002	0.71 (0.57-0.81)	54	88	9	3
Sugar- and fat-rich foods	34 (0-114)	64 (18-148)	–27 (–113 to 29)	<.001	0.43 (0.22-0.60)	40	79	14	7

^a^FFQ: food frequency questionnaire.

^b^WR: weighed food record.

^c^For dietary supplements, n=40 (52%) and n=14 (18%) for DIGIKOST-FFQ and WR, respectively, and Fischer exact test was performed (*P*<.39).

^d^Wilcoxon signed rank test.

**Figure 2:**
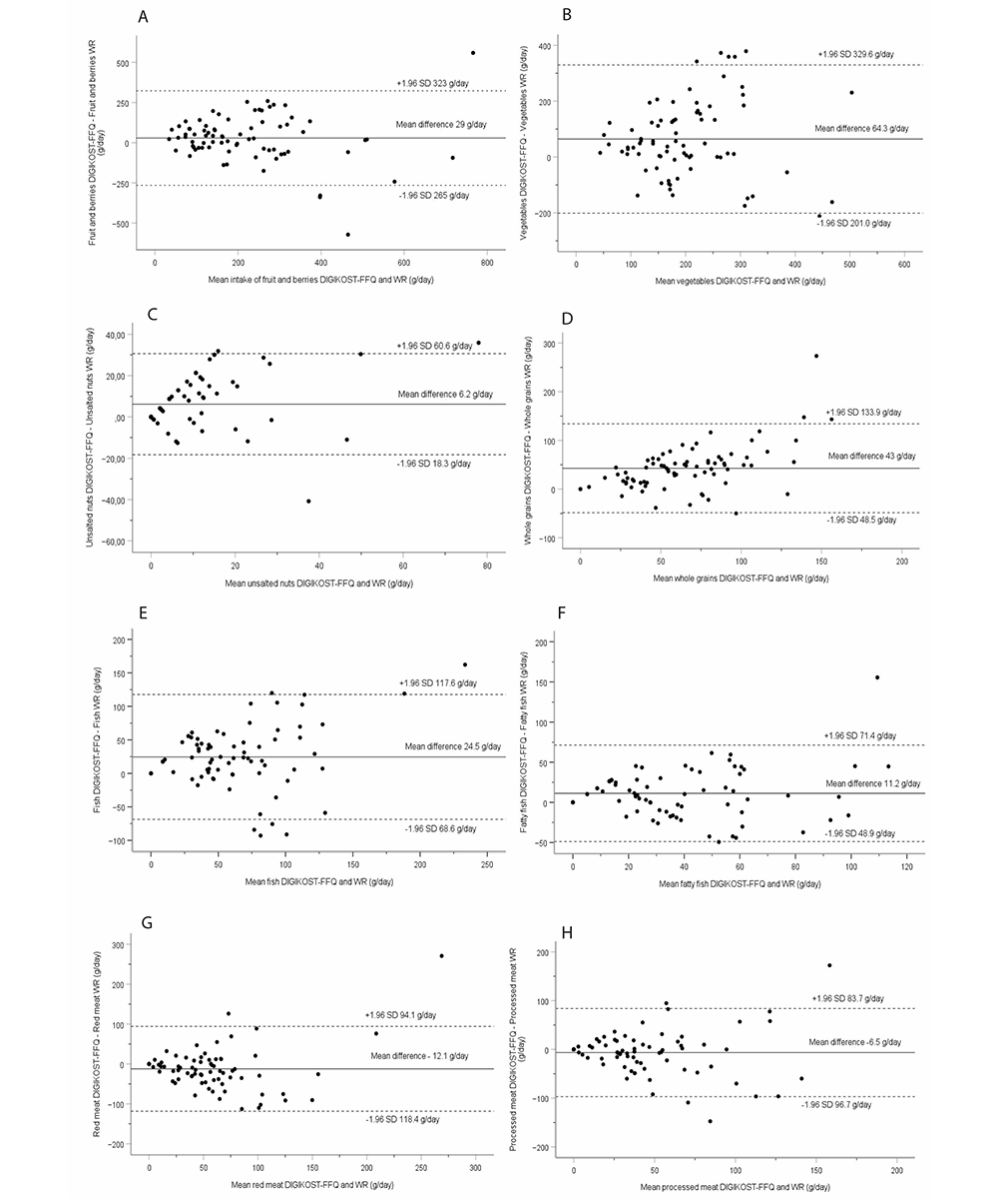
Bland-Altman plots depicting the mean differences (DIGIKOST–WR) for intake of food groups in grams per day: (A) fruits and berries including juice (LoA: –265 to 323), (B) vegetables (LoA: –201 to 329), (C) unsalted nuts (LoA: –18 to 30), (D) whole grains (LoA: –48 to 133), (E) fish (LoA: –68 to 117), (F) fatty fish (LoA: –48 to 71), (G) red meat (LoA: –118 to 94), and (H) processed meat (–96 to 83). The solid line represents the mean, and the dashed lines represent the LoA equal to 1.96 SDs of the observations. FFQ: food frequency questionnaire; LoA: limits of agreement; WR: weighed food dairy.

**Figure 3:**
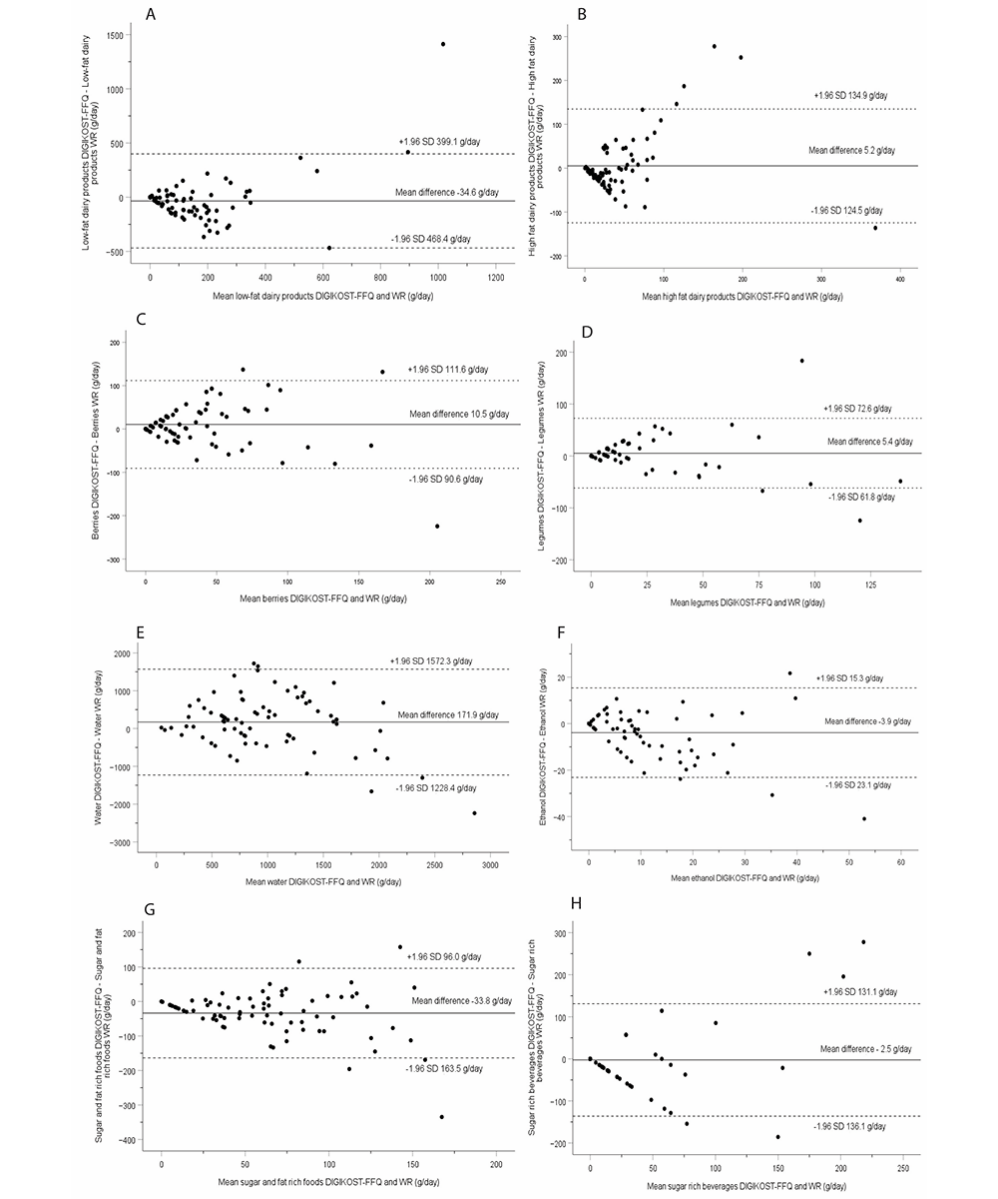
Bland-Altman plots depicting the mean differences (DIGIKOST–WR) for intake of food groups in grams per day: (A) low-fat dairy products (LoA: –468 to 399), (B) high-fat dairy products (LoA: –124 to 134), (C) berries (LoA: –90 to 111), (D) legumes (LoA: –61 to 72), (E) water (LoA: –1228 to 1572), (F) ethanol (LoA: –23 to 15), (G) sugar- and fat-rich foods (LoA: –163 to 96), and (H) sugar-rich beverages (LoA: –136 to 131). The solid line represents the mean, and the dashed lines represent the LoA equal to 1.96 SDs of the observations. FFQ: food frequency questionnaire; LoA: limits of agreement; WR: weighed food dairy.

The median differences were however generally small and below a regular portion size for the different food groups. The ranking of individual intakes was fair and satisfactory for all food groups and poor for “vegetables.” Based on the Bland-Altman plot evaluations, the agreement between the 2 methods was good, with most of the differences within the 95% limit of agreement for each food group and evenly distributed above and below the mean difference (Figures 2 and 3). In total, 69% or more of the participants were classified into the same or adjacent quartile for all food groups, and gross misclassification ranged from 0 for legumes, fatty fish, and alcohol to 13% for unsalted nuts (Table 2). The sensitivity and specificity of the DIGIKOST-FFQ are presented in Table 3. The sensitivity for different food groups ranged from 32% for red meat to 90% for low-fat dairy products. For specificity, the food groups ranged from 40% for unsalted nuts to 100% for sugar- and fat-rich foods. Most of the participants achieved intermediate adherence to the diet score recommendation with both methods (Table 4) [32]. More detailed results are presented in the sections below.

**Table 3:** Proportion of individuals with low adherence (sensitivity) and high adherence (specificity) measured by both assessment methods.

Variables	Sensitivity (low adherence) (%)	Specificity (high adherence) (%)
Fruits and berries	80	70
Vegetables	70	82
Unsalted nuts	82	40
Whole grains	41	85
Fish (lean and fat)	33	87
Red meat (processed and unprocessed)	32	88
Processed meat (white and red)	77	56
Sugar- and fat-rich foods	73	100
Drinks with added sugar	33	87
Low-fat dairy products	90	55
Margarines, butter, and oils	62	66
Ethanol	80	65
Dietary supplements	64	49
MVPA^a^ score^b^	100	65.5

^a^MVPA: moderate to vigorous physical activity.

^b^Fischer exact test (*P*<.4).

**Table 4:** Adherence to composite diet score and MVPA^a^ score (components in the Norwegian lifestyle index [32]).

Component score and assessment tool			Adherence (%)			
			0^b^		0.5^c^	1^d^
**MVPA score**						
	DIGIKOST-FFQ^e^	16.1		19.6		64.3
	SWA^f^	28.6		0.0		71.4
**Diet score**						
	DIGIKOST-FFQ	18.2		66.2		15.6
	WR^g^	10.4		64.9		24.7

^a^MVPA: moderate to vigorous physical activity.

^b^No adherence.

^c^Intermediate adherence.

^d^High adherence.

^e^FFQ: food frequency questionnaire.

^f^SWA: SenseWear Armband Mini.

^g^WR: weighed food record.

#### Fruits, Berries, Vegetables, Whole Grains, and Unsalted Nuts

The DIGIKOST-FFQ slightly overestimated intakes of “fruits and berries” with a median difference of 31 g/day (Table 2). The correlation was satisfactory (*r*=0.64), and 87% of the individual intakes were classified in the correct or adjacent quartile, and only 1% was grossly misclassified. For intakes up to 200 g/day, the limits of agreement were 200 g above and below the mean difference, which increased with higher intakes (Figure 2A). “Vegetables” were overestimated with 45 g/day and with a similar distribution of limits of agreement as for “fruits and berries” (Figure 2B). In total, 69% of the individual intakes of “vegetables” were classified in the correct or adjacent quartile, and 10% were grossly misclassified. The ranking of individual intakes of “vegetables” was poor (*r*=0.24). The median difference for intakes of “whole grains” was 43 g/day, the correlation was satisfactory (*r*=0.51), and 81% of the participants were classified in the same or adjacent quartile, whereas 1% was grossly misclassified. Limits of agreements ranged from –48 to 133 g/day, and the plot indicated a trend for overestimation with increased intakes (Figure 2D). “Unsalted nuts” were estimated with a median difference of 2.3 g/day and with a borderline satisfactory correlation (*r*=0.48). In total, 74% of the participants were classified in the same or adjacent quartile, and 13% were grossly misclassified (Table 2). The distribution of the differences increased with higher intakes, and the limits of agreement were wide (Figure 2C).

#### Fish, Dairy Products, and Meat

Median differences for “fish” and “fatty fish” intakes were 22 and 10 g/day, respectively (Table 2). Ranking of individual intakes was satisfactory with correlations of *r*=0.52 and *r*=0.61 for “fish” and “fatty fish,” respectively. Limits of agreements ranged from –68 to 117 g/day, and the distribution of the differences increased with higher mean intakes up to about 100 g/day of “fish.” For “fatty fish,” the agreement was good with evenly distributed differences above and below the mean difference and narrow limits of agreement (–49 to 71 g/day; Figure 2E and F). Classification of individual intakes was good for both “fish” and “fatty fish” with low gross misclassification (Table 2).

The DIGIKOST-FFQ showed a minor underestimation of dairy products on the group level, acceptable ranking (*r*=0.65), low gross misclassification of individual intakes, and good agreement between the methods (Table 2 and Figure 3A and B). The DIGIKOST-FFQ was able to estimate intakes of “red meat” and “processed meat” on the group level and to rank individual intakes of both food groups (Table 2 and Figure 2G and H). More than 82% of the participants were classified in the same or adjacent quartile, and few were grossly misclassified.

#### Sugar- and Fat-Rich Foods and Beverages, Other Beverages, and Dietary Supplements

There were good agreements between the methods in estimating intakes of “sugar- and fat-rich foods” and “sugar-rich beverages” on the group level, fair ranking of individual intakes, and good quartile classifications (Table 2 and Figure 3G and H). Intakes of “water” were overestimated by the DIGIKOST-FFQ, and the ranking of individual intake was fair (Table 2 and Figure 3E). The DIGIKOST-FFQ was able to estimate the intake of “ethanol” both on group and individual levels (Table 2). More than 79% of the participants were classified in the same or adjacent quartile for intakes of “sugar- and fat-rich foods,” “sugar-rich beverages,” “water,” and “ethanol” (Table 2).

### Physical Activity, Sleep, and Sedentary Time

**Figure 4:**
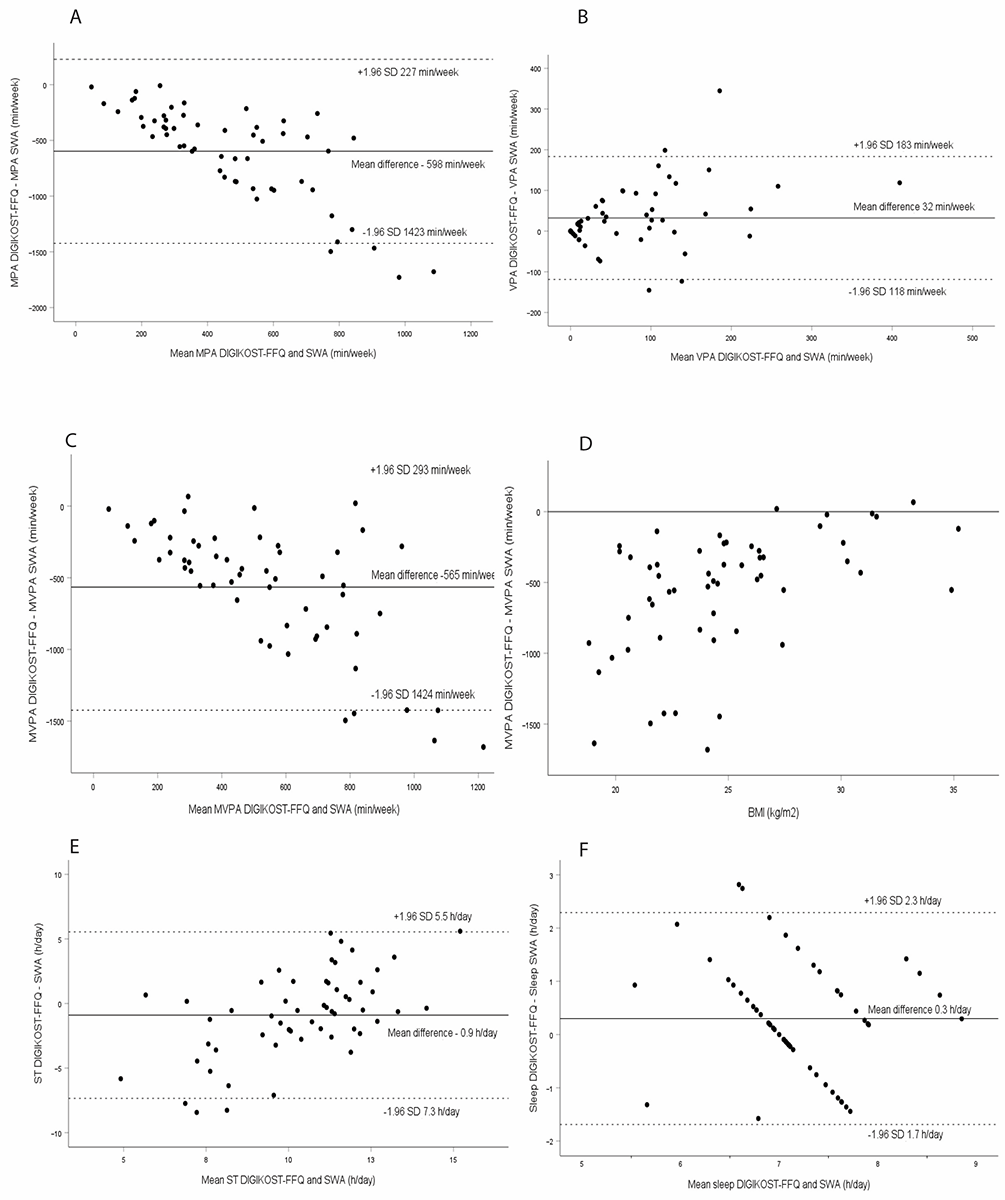
Bland-Altman plots depicting the mean differences (DIGIKOST–activity sensor [SWA]) for activities in minutes per week: (A) MPA (LoA: –1423 to 227), (B) VPA (LoA: –118 to 183), (C) MVPA (LoA: –1424 to 293), (D) BMI, (E) ST (LoA: –7 to 6), and (F) sleep (–1.7 to 1.9). The solid line represents the mean, and the dashed lines represent the LoA equal to 1.96 SDs of the observations. FFQ: food frequency questionnaire; LOA: limits of agreement; MPA: moderate physical activity; MVPA: moderate to vigorous physical activity; ST: sedentary time; SWA: SenseWear Armband Mini; VPA: vigorous physical activity.

The DIGIKOST-FFQ underestimated time in moderate intensity (median –468 min/week), which increased with higher amounts of time in moderate physical intensity (Table 5 and Figure 4A). The correlation between the methods was borderline fair (*r*=0.29; Table 4). In total, 73% of the participants were classified in the correct or adjacent quartile, and 5% were grossly misclassified. On the group level, vigorous intensity was overestimated by 18 min/week, and the individual differences increased with increased time in “vigorous physical activity” (Table 5 and Figure 4B). There was a fair correlation between the methods for vigorous intensity (*P*=.40), and no gross misclassification. The DIGIKOST-FFQ was able to rank individual time in “moderate to vigorous intensity” (*P*=.64), and 79% were classified in the correct or adjacent quartile, and few were grossly misclassified (Table 5). On the group level, time in “moderate to vigorous intensity” was underestimated by DIGIKOST-FFQ, which also increased with higher amounts of time in this intensity (Table 5 and Figure 4C). The underreporting of “moderate to vigorous intensity” was particularly seen among individuals with BMI <25 kg/m^2^ (Figure 4D).

**Table 5:** Estimated physical activity, sedentary time, and sleep from DIGIKOST-FFQ^a^ and SWA^b^ for all participants (n=56).

Physical activity intensity	DIGIKOST-FFQ, median (P10-P90)	SWA, median (P10-P90)	Difference, median (P10-P90)	*P*value^c^	Spearman ρ (95% CI)	Correct quartile	Correct+adjacent quartile (%)	Misclassification 2 quartiles (%)	Grossly misclassification (%)
MPA^d^ (min/week)	123 (33-428)	753 (247-1492)	–468 (–1333 to –156)	<.001	0.29 (0.02 to 0.50)	30	73	21	5
VPA^e^ (min/week)	62 (0-226)	21 (0-178)	18 (–42 to 123)	<.001	0.64 (0.45 to 0.78)	43	82	18	0
MVPA^f^ (min/week)	236 (48-507)	754 (258-1534)	–453 (–1424 to 123)	<.001	0.40 (0.14 to 0.60)	34	79	16	5
Sedentary time (not sleep; h/day)	10 (5-14)	11 (8-13)	–0.63 (–6 to 3)	.06	0.27 (–0.001 to 0.5)	41	75	16	9
Sleep (h/day)	7 (7-8)	7 (6-8)	0.21 (–1.3 to 1.7)	.03	0.19 (0.08 to 0.43)	36	71	20	9

^a^FFQ: food frequency questionnaire.

^b^SWA: SenseWear Armband Mini.

^c^Wilcoxon signed rank test.

^d^MPA: moderate physical activity.

^e^VPA: vigorous physical activity.

^f^MVPA: moderate to vigorous physical activity.

The DIGIKOST-FFQ was able to estimate time in “sedentary” activities on the group level, with a trend for overreporting with higher amounts of time in this activity, and the correlation between the methods was poor (*r*=0.27; Table 5 and Figure 4E). A high number of participants (75%) were classified in the correct or adjacent quartile, and 9% were grossly misclassified. Median time in “sleep” was estimated to be 7 hours per day with both methods, with a trend for underreporting by the DIGIKOST-FFQ with increased time in “sleep” (Table 5 and Figure 4F). The correlation was poor (*r*=0.19), but the classification of individual time in quartiles was good (71%; Table 5).

### Adherence to Dietary Guidelines and Physical Activity Recommendations

The sensitivity was above 60% for all dietary guidelines except for “whole grains,” “fish,” “red meat,” and “drinks with added sugar” (Table 3). The specificity was above 60% for all dietary guidelines except for “unsalted nuts,” “processed meat,” “low-fat dairy products,” and “dietary supplements” (Table 3). The total mean diet score of the Norwegian diet index (ranging from 0 to 20 points) was estimated to be 11.2 (SD 3.2) and 10.4 (SD 3.1) points with the DIGIKOST-FFQ and WR, respectively (Multimedia Appendix 2) [32]. Adherences to each component in the Norwegian diet index measured by DIGIKOST-FFQ, WR, and SWA are shown in Tables 4 and 6. Intermediate adherences were the most frequent for the composite “diet score,” “fruits and berries,” “vegetables,” and “margarine, butter, and oils” estimated with both DIGIKOST-FFQ and WR. Low adherences were shown by both methods for “unsalted nuts,” “processed meat,” and “sugar- and fat-rich foods,” whereas high adherences were shown for “fish,” “sugar-rich drinks,” and “dietary supplements.” Approximately, evenly distributed adherences (ie, low, intermediate, and high) with both methods were shown for “red meat” (Table 6). High adherence to the recommendation of physical activity was estimated as the most frequent by both methods (Table 4).

**Table 6:** Adherence to the Norwegian diet index [32].

Food component and dietary assessment tool		Adherence (%)		
		0^a^	0.5^b^	1^c^
**Fruits and berries**				
	DIGIKOST-FFQ^d^	24.7	40.3	35.1
	WR^e^	37.7	32.5	29.9
**Vegetables**				
	DIGIKOST-FFQ	16.9	37.7	45.5
	WR	41.6	44.2	14.3
**Unsalted nuts**				
	DIGIKOST-FFQ	63.6	16.9	19.5
	WR	81.8	11.7	6.5
**Whole grains**				
	DIGIKOST-FFQ	11.7	24.7	63.6
	WR	45.5	37.7	16.9
**Fish**				
	DIGIKOST-FFQ	13.0	10.4	76.6
	WR	28.6	22.1	49.4
**Red meat**				
	DIGIKOST-FFQ	19.5	29.9	50.6
	WR	36.4	32.5	31.2
**Processed meat**				
	DIGIKOST-FFQ	66.2	13.0	20.8
	WR	67.5	10.4	22.1
**Margarine or oils**				
	DIGIKOST-FFQ	13.0	37.7	49.4
	WR	26.0	35.1	37.7
**Dairy**				
	DIGIKOST-FFQ	39.0	23.4	37.7
	WR	24.7	14.3	61
**Foods high in sugar and fat**				
	DIGIKOST-FFQ	64.9	10.4	24.7
	WR	89.6	7.8	2.6
**Sugar-sweetened beverages**				
	DIGIKOST-FFQ	20.8	0	79.2
	WR	3.09	6.5	54.5
**Dietary supplements**				
	DIGIKOST-FFQ	53.2	—^f^	46.8
	WR	18.2	—	81.8

^a^No adherence.

^b^Intermediate adherence.

^c^High adherence.

^d^FFQ: food frequency questionnaire.

^e^WR: weighed food record.

^f^Not available.

## Discussion

### Principal Findings

We investigated the relative validity of the new digital assessment tool, DIGIKOST-FFQ, with 7-day WR and activity sensors as reference methods. Generally, the DIGIKOST-FFQ was able to rank individual intakes of foods and time spent in physical activities adequately and also able to evaluate dietary intakes and adherences to the Norwegian FBDG and physical activity recommendation.

The DIGIKOST-FFQ is based on the paper form NORDIET-FFQ, and improvements were made to enhance the quality of the DIGIKOST-FFQ estimates. For example, in the DIGIKOST-FFQ, questions about fruits and berries were presented as single food items as opposed to aggregated questions in the paper FFQ, which underreported the fruit, berry, and vegetable intakes [26]. In this validation study, improved correlations and agreement between the test and reference methods for estimated fruit, berry, and vegetable intakes were observed. Moreover, WR has been documented to underreport energy and food intake [46,47]. Thus, the overreporting of fruit and vegetable intakes in the DIGIKOST-FFQ along with the improved correlations and agreement between the methods in this validation study indicate a higher accuracy of the intake estimates from the DIGIKOST-FFQ for these food groups as compared to the NORDIET-FFQ. In particular, the DIGIKOST-FFQ showed a borderline fair correlation in ranking individual intakes of vegetables. However, the median difference between the methods was well below 1 portion of vegetable (ie, 100 g), the agreement between the methods was good, and the DIGIKOST-FFQ was able to identify adherence to the recommendation of vegetables.

Few participants reported intakes of unsalted nuts, and low amounts were reported for those with intakes. This may explain the high share grossly misclassification of nut intakes in this study.

Adding single-item questions regarding whole grain products along with automatic counting of slices of bread consumed per week to help the responder to report intakes of bread in the DIGIKOST-FFQ (Multimedia Appendix 1) may have successfully contributed to the improvement of the reported intakes of whole grains. In addition, legumes were added as a new item in the DIGIKOST-FFQ, and the good agreement and correlation observed for intakes of legumes contributed to the overall good ability of DIGIKOST-FFQ to identify individuals with different adherences to the recommendations for all fiber-rich foods.

Reported intakes of fish, meat, dairy products, sugar- and fat-rich foods, sugar-rich beverages, ethanol, water, and dietary supplements were good with the DIGIKOST-FFQ. We hypothesize that the overreporting of water, corresponding to 1 glass of water per day, might be explained by the warmer climate in June 2021, during which most of the participants completed the DIGIKOST-FFQ, as compared to the colder climate in August 2021 when they completed the WR, thus resulting in higher intakes of water in June.

Underreporting of physical activity by questionnaires is a common issue when compared to objective activity sensors, which record all activities within 24 hours a day, whereas questionnaires rely only on a few questions supported by others [48,49]. In particular, sex and BMI have been shown to alter the association between self-reported and objectively measured physical activity [49]. Additionally, questionnaires are subjected to memory and conceptualization of different degree of intensities, such as the difference between moderate and vigorous intensities. In this study, there was a trend for normal-weighed individuals to underreport moderate physical activity more than overweighed or obese individuals. Overall, on the group level, the DIGIKOST-FFQ underreported moderate to vigorous physical activity by 450 minutes per week when compared to SWA. In particular, low and moderate intensities of physical activity, such as transport to work (eg, biking), gardening, and housework, have been challenging to report in questionnaires [50,51], which was also observed in this study. Moreover, the aim of the DIGIKOST tool is to estimate adherence to the recommendations and to give advice in the individual report in order to spend more time in physical activity. Therefore, ranking individual time in different activities and classifying the individuals according to adherence are important abilities of the assessment tool.

Adding illustrations of different intensities with images and text for the participants to better differentiate between the performance of physical activity during the day and week may increase the quality of the estimated time in moderate physical activity. Generally, the results from this study showed that the DIGIKOST-FFQ was able to rank individual dietary intakes and identify adherences to most of the dietary guidelines and physical activity recommendations. This conforms to previous studies showing the ability of FFQs to classify individuals according to different intakes of foods and time in activities [8,9].

### Strengths and Limitations

We included 77 participants in the study, which is an adequate number in validation studies. The announcement by email lists from the National Registry of Norway was accompanied by an advertisement in Facebook. Recruitment through Facebook has shown to be the most successful strategy. Generally, the use of social media for recruitment in health research has shown to be effective in hard-to-reach populations [52,53]. In this study, most of the participants were women with higher education than the general Norwegian population [54]. This might compromise the external validity of this study.

In this study, the DIGIKOST-FFQ (test method) was administered prior to the reference methods in order to avoid the learning effect from the reference method [11]. Moreover, the DIGIKOST-FFQ asks for dietary intakes and activities the last 2 months, whereas the reference methods (WR and SWA) prospectively recorded dietary intake and activities for 7 consecutive days and completed within 1-2 months after fulfilling the DIGIKOST-FFQ. The average range of days between completion of DIGIKOST-FFQ and reference methods was 19 (SD 16.8) days. The expectancy of meaningful changes in dietary intakes or activities during this period is low.

The measurement errors usually associated with printed questionnaires have also been documented in digital questionnaires such as underreporting and recall errors [6,10,12,26,31,41,55,56]. Closed questions, predefined categories of frequency and amounts, and foods not included in the questionnaire may result in misclassification and underreporting. In DIGIKOST-FFQ, underreporting was documented for some food groups and activities, but there were few gross misclassifications, which indicates the good ability to identify individuals with different adherence to the recommendations. Ideally, in validation studies, the error in the reference method should be independent from the test method [16]. This study used a self-reported instrument, that is, the WR, as the reference method against the DIGIKOST-FFQ. Since both methods are self-reported, the errors may not be independent, thus inflating the validity correlation coefficient and an optimistic evaluation of the validity of the FFQ. Even though the WR is commonly used as a reference method in the validation of FFQs [11,12] and does not rely on the memory of food intakes, no interpretation of portion size is needed due to the weighing. Moreover, the WR provides data on foods that are really consumed, which are not provided by using biomarkers as a reference method. The DIGIKOST-FFQ does not cover a whole diet, since the aim of the tool is to estimate adherence to the recommendations, thus biomarkers for energy intake or nutrients are not used in this study. In the future, revisions of the DIGIKOST tool developments may be conducted to enable estimations of energy and food intakes; however, that is not the design and aim of this version.

### Conclusions

The DIGIKOST-FFQ was able to assess dietary intake and physical activity and identify individuals with different adherences to the Norwegian FBDG and physical activity recommendation. However, moderate physical activity was underreported, water was overreported, and vegetables showed a low correlation between the methods, which is important to take into account when interpreting the data in future studies. The new digital questionnaire contributes with valuable data for research projects focusing on improved lifestyle, prevention of diseases, and increased quality of life and survival, as well as a screening tool in the clinic.

## Appendix

Multimedia Appendix 1 DIGIKOST: a digital application for diet and lifestyle assessment and benchmarking against national guidelines.

Multimedia Appendix 2 Adherence to total diet score and each single component measured with both assessment tools.

## Abbreviations

FBDG: food-based dietary guidelines
FFQ: food frequency questionnaire
SWA: SenseWear Armband Mini
WR: weighed food record
