# Supplementary material for: Digital Food Frequency Questionnaire Assessing Adherence to the Norwegian Food–Based Dietary Guidelines and Other National Lifestyle Recommendations: Instrument Validation Study
Source: J Med Internet Res. 2024 Apr 30;26:e53442. doi: 10.2196/53442 (PMC11094607; doi:10.2196/53442)
Supplement: Multimedia Appendix 1 [file jmir_v26i1e53442_app1.pdf]

DIGIKOST: a digital application for diet and lifestyle assessment and benchmarking against national guidelines

We have developed a digital diet and lifestyle assessment tool; the DIGIKOST application, including several tools and technical functions, i.e. DIGIKOST-FFQ, DIGIKOST dataset and the DIGIKOST report. The DIGIKOST-FFQ assess dietary intake (i.e. foods, but not nutrients and energy intake) and other lifestyle factors according to the Norwegian FBDG, whereas the DIGIKOST report is an individual feedback report on respondent's adherence to the Norwegian FBDG, with specific and personalized advices on how to fulfill the recommendations.

Software platform

The DIGIKOST-FFQ is based on a software platform called Nettskjema, developed and administered by University Information Technology Center (USIT) at University of Oslo. Norway [57]. Nettskjema is a digital tool used for collecting sensitive data in research projects both in the form of online questionnaires or as mobile apps. Respondents can submit answers from a browser on a computer, mobile phone or tablet. It is applicable for use in large-scale surveys and is easy and feasible in use for the respondents. When Nettskjema is connected to the Services for Sensitive Data (TSD), it is regarded as the most secure application in Norway to be used in research [33]. TSD is part of the national e-infrastructure for research operated by Sigma2, enables secure handling, transfer and sharing of data between researchers, and is administered by USIT. Research projects are issued a storage space and access to a large array of relevant software in Windows (SAS, Matlab, stat, R etc.) or Linux (Lible, Office, R etc.) servers. The DIGIKOST-FFQ is connected to TSD, to which all data are sent directly and safely stored.

The main login option in DIGIKOST is the ID-port (e-ID used by the Norwegian Agency for Public Management and eGovernment (Difi)) [29, 30], and in order to receive the DIGIKOST report the participant is obligated to log in with this function. Another login function to complete the DIGIKOST-FFQ, although less secure, is to type the national ID number at the first page of the DIGIKOST-FFQ. All researchers using the DIGIKOST application must apply for and create a study project in TSD Researchers connected to the project will get access to their TSD project through the ID-port function.

Development of DIGIKOST

The DIGIKOST-FFQ

The DIGIKOST-FFQ is based on the NORDIET-FFQ, a paper-based, validated, short, semi-quantitative food frequency questionnaire developed at the Department of Nutrition, University of Oslo [26, 31]. Based on previous validation studies of the NORDIET-FFQ [26, 31], we did the following adjustments in the DIGIKOST-FFQ:

1. The questions about intake of fruit in the NORDIET-FFQ were changed from asking about intakes of small-, medium- or large sized fruits into the most common eaten fruit species in each category, such as apple/pears, orange, banana, plums and grapes in the DIGIKOST-FFQ.
2. Dried fruit did not contribute to overall fruit intake, therefore this item was removed in the DIGIKOST-FFQ.
3. In the NORDIET-FFQ intake of vegetables was under-reported and ranking of study participants based on intake of vegetables was poor [26]. The original aggregated question regarding "Other vegetables", including many commonly eaten vegetables,

was changed in the DIGIKOST-FFQ into separate questions for each item, such as carrots, broccoli and root vegetables.

4. A question regarding intakes of legumes was added.
5. Intake of whole grains was over-reported in the NORDIET-FFQ [26]. Changes were made in DIGIKOST-FFQ aiming to help individuals to report their intakes of whole grains products more accurately, like distinguishing between bread with different contents of whole grain, as well as implementing an automatic function to calculate number of slices of bread, images of portion sizes and weight measurements in grams.
6. Questions regarding porridge were added in the DIGIKOST-FFQ.
7. Underreporting of dairy products and difficulties in interpreting questions on dairy consumption were found in NORDIET-FFQ [26]. Therefore, images of portion sizes and weight measurements in grams or household measures were added in DIGIKOST-FFQ. In addition, yoghurt was separated as a single category.
8. In the DIGIKOST-FFQ, images of portion sizes and weight measurements in grams were added for questions regarding fish and meat products.
9. Sedentary time was under-reported in the NORDIET-FFQ [31], therefore this activity was differentiated into single questions regarding time being sedentary during work and during leisure time.
10. A question about sleep was also added in the DIGIKOST-FFQ.

Furthermore, portion sizes of some specific foods were adjusted according to an updated report on standard portion sizes published in 2015 [34].

The DIGIKOST-FFQ assesses data on single food items as well as food groups according to the Norwegian FBDG and the data is presented in grams per day (Table S1). The DIGIKOST dataset also contains variables estimating amounts and time spent on physical activity and sedentary time, alcohol- and tobacco use, body weight and height as well as demographic data (Table S2). Overall, it includes 103 food and lifestyle items, of which 78 questions about food items are condensed into 14 main food groups, 7 questions about physical activity, sedentary time and sleep, 8 questions about tobacco use and 10 questions about body weight and demographic data. As the DIGIKOST estimate intakes of foods according to the dietary guidelines and thus not a whole diet, nutrients and energy intake cannot be estimated. Design view of DIGIKOST-FFQ in printed version (only for demonstration for this paper) can be found in Figure S2.

The automatically generated dataset retrieved from the DIGIKOST-FFQ also presents variables of different food groups according to the recommendations (Table S3). In order to characterize the subjects` adherence to the Norwegian FBDG, the demographic data are fundamental, because the recommendations vary according to the demographics. For instance, when characterizing the intake of whole grains, there are different recommendations based on gender (Tables S2 and S3). Further details regarding the principles of combining different foods and the cut-off values conforming to the recommendations are thoroughly described in Henriksen et al. [26, 32, 27].

The DIGIKOST-FFQ includes three items regarding alcoholic beverages regularly used in Norway; beer, wine and spirit (Table S2). The other lifestyle components included in the DIGIKOST-FFQ (Table S2), corresponds to the lifestyle recommendations of the Norwegian health authorities [32, 35, 36] with a priori defined limits and cut-off values of adherence (Table S4).

The recommendations of physical activity focus on performing moderate- to vigorous intensity physical activity for at least 150 minutes per week [32, 35, 3]. Therefore, the questions regarding physical activity in the DIGIKOST-FFQ ask for moderate and vigorous intensity only, and not light intensity. Attaining a healthy weight is shown to promote beneficial health outcomes and is defined as having body mass index (BMI, kg/m^2) within normal range (i.e. 18.5-24.9 kg/m^2) [37] (Table S3). Two items in the DIGIKOST-FFQ cover this recommendation when asking for body weight in kilograms and height in cm (Table S2).

The DIGIKOST software platform
We developed a software platform were participants used an ID-port log in function linked to in the invitation. The next step for the invitee is to read the informed consent and confirm their participation by an online signature. After the participant has confirmed his/her participation, the filling in of the DIGIKOST-FFQ start. After the respondent's completion of the FFQ, the DIGIKOST report becomes available by the automatic receipt function and can be read on the electronic device or printed out as a pdf-file (Figure S1).

Secure handling of data
The development of DIGIKOST has been conducted in a close cooperation with the University Center for Information Technology (USIT) at UiO and all data registered and generated by the systems are stored in the secure server, TSD, situated at USIT/UiO (blue circles in Figure S1). The DIGIKOST dataset is available as CSV files and MS Excel files and the DIGIKOST report is available for the researchers or clinicians responsible for the data collection and with access to the specific TSD project (Figure S1 and Table S6).

A new front-end application
We have developed a new front-end application (i.e. a user interface), which automatically counts the number of reported slices of bread reported and then visualize these in "real time" for the user in the DIGIKOST-FFQ. This sum of bread slices is also presented in the helping text in the section with questions about different spreads (Figure S2 and Table S6). Also, helping the participants to register portion sizes is the inclusion of high-quality images of portion sizes and illustrations of amount for different foods, together with text explaining how to report dietary intake and other lifestyle factors. The skip algorithms used for the introductive questions on whether or not the respondent has a usual intake (i.e. at least once per week) of the food or performing a lifestyle activity regularly, aim to decrease the time of completion of the DIGIKOST-FFQ (Figure S2 and Table S6).

The new algorithms
We also developed an algorithm that convert the DIGIKOST-FFQ responses to assessment values relevant for national guidelines. This allowed us to benchmark the assessment values to national guidelines operationalized by Henriksen et al. [26], and to develop a report that present the individual benchmarked assessment results.

The algorithms automatically transform the crude variables generated from the standard code-book in the Nettskjema [38] into dietary and lifestyle variables described in Tables S1, S2, S3 and S4. The DIGIKOST-FFQ contains questions on both frequency and amounts for each food item or activity. The options of frequency of intake for different foods or frequency of physical activity vary from times per day to seldom/never. Portion sizes or amounts per time are specified for each item and include different ranges in amounts. The algorithms calculates intakes or activity in grams/minutes/hours per day or week by multiplying frequency and amounts for each item. Moreover, the algorithms also aggregates all single items contributing

to a specific food group. For instance, the food group called “fruits” is the sum of intakes of several single fruit-items, such as apples and pears, orange, banana, small fruit, berries and one glass of juice (Table S1). Thus, the algorithms predefine the different combinations of single items needed to make all the variables in the dataset (Figure S1, Tables S1-S4). The algorithms are coded as a R-script package and, together with the dataset, saved in TSD [57] (Figure S1).

The digital assessment report (DIGIKOST report)
After completion of the DIGIKOST-FFQ, the participants automatically receive feedback related to their individual adherence to the dietary and lifestyle recommendations, followed by advices on how to improve their lifestyle (Figures S1 and S3).

The algorithms used to generate the DIGIKOST report (Figure S1) are based on a three level set of rules for each recommendation, reflecting different levels of adherence to the recommendations and further described in Henriksen et al. [32]. Pre-defined cut-off values for each recommendation correspond to the three levels of adherence, 1) no adherence (0 point), 2) intermediate adherence (0.5 point) and 3) high adherence (1 point). Each level of adherence is associated with an advice to the participant. For instance, the recommendation for intake of fruit, high adherence (i.e. 1 point) will result in the following advice: “You follow the recommendation for fruit intake. Your fruit intake is 250 (an example) grams per day. Keep up the good work, this is beneficial for your health.” If the participants reach a score of intermediate adherence, the advice is “You partially follow the recommendation for fruit intake. Your fruit intake is 140 (an example) grams per day. It is great that you eat some fruit, however, if you increase your intake with for instance one banana and a plum per day you will reach the recommended daily intake”. And, if the participants score no adherence the advice is: “You do not follow the recommendation for fruit intake. Your fruit intake is 80 (an example) grams per day. If you increase the intake with for instance one banana, a plum and a handful of berries per day you will reach the recommended daily intake” (Figure S3).

Improvements based on focus groups interviews
The first draft of DIGIKOST-FFQ was found easy to use and feasible in focus groups interviews [27]. However, some issues were identified for further improvement:

1. the completion time of the questionnaire should not take more than 20 minutes
2. participants found it difficult to report dietary intake, sedentary time and physical activity last year due to seasonal variation
3. pictures with portion size were helpful, but some portion sizes were difficult to differentiate between
4. the graphical presentation of the degree of adherence to the Norwegian FBDG in the DIGIKOST report was difficult to interpret,
5. the participants found it difficult to interpret their adherence to the recommendations when results were presented as health index

On average, the DIGIKOST-FFQ takes about 20 minutes to complete [27]. However, since many food categories have introductive questions with automated skip algorithms, most people will use shorter time to complete the questionnaire.

Based on the focus group results we revised the DIGIKOST-FFQ as described in Table S5. In particularly, the timeframe for reporting of diet in the DIGIKOST-FFQ was changed to “within the last two months”. We also added text prior to new food groups, added grams as reference values to the images of portion sizes and added some questions on missing food items. The main revisions of the DIGIKOST report included removal of the sections found to

be challenging to interpret for the participants, such as the graphical presentation of adherence as well as a health index (Table S5).

Based on the results from the usability test [27], we decided to remove several sections in the report which the participants found challenging to understand and interpret. However, the table with dietary intake and physical activity compared to the Norwegian FBDG was included in the beginning of the DIGIKOST report. Moreover, the individual benchmarking against the Norwegian FBDG with individual advice in how to fulfil these recommendations, were presented in the last section of the report (Figure S3).

Abbreviations

Norwegian FBDG: Norwegian food-based dietary guidelines
FFQ: food frequency questionnaire
USIT: University Center for Information Technology
UiO: University of Oslo
TSD: Services for sensitive data (Tjenester for Sensitive Data)
BMI: Body mass index

Table S1: Dietary variables included in the DIGIKOST dataset

**Food group**	**Food items**
Fruits, berries	Apples and pears, orange, banana, small fruit (plums, grapes, kiwi), berries (blueberry, strawberry, cloudberry), 1 glass of juice
Vegetables	Carrot, broccoli, cauliflower, tomato, tomato products, onion/leek, mixed salad, root vegetables (radishes, rutabaga, celery, parsley root), avocado, cucumber, zucchini/squash, eggplant
Nuts	Salted nuts (peanuts, chili nuts, nut mix, cashew nuts) and unsalted nuts (almonds, walnuts, cashew nuts, nut mix, peanuts)
Legumes	Beans, peas, lenses, chickpeas
Whole grain products	Bread with 25-50% wholemeal flour (60 % cereals), bread with 50-75 wholemeal flour (60 % cereals), bread with 75-100% wholemeal flour (60 % cereals), wholemeal crisp bread, unsweetened cereals, oatmeal porridge, brown rice, wholegrain pasta
Refined grain products	Sweetened cereals (e.g. Corn Flakes), rice porridge, white rice, white pasta, cakes, buns, waffles, sweet biscuit
Fish	Fatty fish (e.g. salmon, trout, herring, halibut), lean fish (e.g. cod, pollock, angler), processed fish (e.g. fish gratin, fish cakes) (40 % fish)), fish as spread (e.g. mackerel, smoked salmon, herring)
Red meat	Beef, pork, lamb and goat: non-processed red meat, processed red meat, red meat as spread
Poultry/white meat	Non-processed white meat, processed white meat, white meat as spread
Processed meat	Processed red meat, processed white meat, red meat as spread, white meat as spread
Oils and margarines	Oils and margarines High content of unsaturated fatty acids
Butter	Butter with great proportion of saturated fatty acids
Low-fat dairy products	Low-fat dairy products (lean sour crème, lean crème fraiche), lean yoghurt, fat-reduced cheese, lean milk
Dairy products that contain high levels of saturated fat	High-fat dairy products, high-fat cheese, yoghurt, whole milk
Water	Water from tab or bottle
Drinks with added sugar	Syrup, sugar-sweetened soft drinks, iced tea with sugar
Drinks without added sugar	Artificially sweetened soft drinks, artificially sweetened ice tea, artificially sweetened syrup
Juice	Apple juice, orange juice without added sugar
Coffee and tea	Filter coffee, boiled coffee/coffee capsules, espresso, cappuccino, cafe latte, macchiato, instant coffee, black and green tea
Foods with high content of sugar and fat	Cakes, buns, waffles, sweet biscuit, dessert, chocolate, sweet candy, chips, sugar-rich spread (e.g. honey, jam, peanut butter etc.)
Dietary supplements	Cod liver oil, cod liver oil capsules, fish oil capsules, omega-3 supplements, vitamin D, multivitamin, iron, Calcium

Table S2: Lifestyle and demographic variables included in the DIGIKOST dataset

**Lifestyle/demographic group**	**Lifestyle/demographic items/units**
Age	Years
Gender	Man/Women
Weight	Kg
Height	Cm
Physical activity	Moderate intensity: 3-6 METS: Times per week and amounts in minutes per time<br>Vigorous intensity: ≤ 6 METS: Times per week and amounts in minutes per time
Sedentary time	Hours<br>Sedentary time at work<br>Sedentary time during leisure time<br>Sleep
Alcohol	Beer, wine, spirit
Smoking/use of snuff	Non-smoker/non user of snuff<br>Seldom smoker/ user of snuff<br>Daily/regular smoker/ user of snuff<br>Former smoker/ user of snuff<br>If smoker/ user of snuff or quit smoking/ using snuff: how many cigarettes/boxes with snuff per day/week<br>If smoker/ user of snuff or quit smoking/using snuff: How many consecutive years as smoker/ user of snuff
Living status	Living with others<br>Living alone
Education	Primary school, secondary upper school, high school, University/College of less than or more than 4 years, trade certificate
Working status	Working/employed (partial, full time), not working/home self-chosen, retired, unemployed, sick-leave, rehabilitation, disability benefit, student
Ethnicity of your father	Europe, Africa, Asia, other
Ethnicity of your mother	Europe, Africa, Asia, other
Lived in Norway	Years

Table S3: Operationalization of the dietary recommendations according to the Norwegian food based dietary guidelines included in the DIGIKOST dataset, and a priori defined limits and cut-off values of compliance according to Henriksen et al. [31, 26, 32].

	Dietary recommendations	Intake required to fulfil the recommendations
1.	It is recommended to eat at least five portions, corresponding to at least 500 grams altogether, of vegetables, fruit and berries every day	≥ 500 g/d
2.	About half of this intake should be in the form of fruit and berries	≥ 250 g/d
3.	About half of this intake should be in the form of vegetables	≥ 250 g/d
4.	Eat at least four portions of whole grain products every day. Four portions of wholegrain products corresponds to about 70-90 grams of wholegrain per day	Women: ≥ 70g/d<br>Men: ≥ 90g/d
5.	At least half of the total consumption of grain products should be in the form of whole grains	Whole grains (g/d) > 50% of total grains (g/d)
6.	Reduce cereals with high content of fat and sugar	≤ 20 g/d
7.	Weekly consumption of 300-450 grams of fish is recommended	≥ 43 g/d<br>(300 g/week)
8.	It is recommended that at least 200 grams of the intake should be of fatty fish	≥ 29 g/d
9.	Limit the consumption of red meat (beef, pork, lamb and goat) to 500 grams per week	≤ 71 g/d
10.	Moderate consumption of non-processed meat can be included in the diet	≤ 20 g/d
11.	Preference should be given to the consumption of non-processed meat.	Non-processed meat (g/d) > 50% of processed meat (g/d)
12.	Limit the intake of processed meat	≤ 20 g/d
13.	Reduce intake of foods with high content of sugar and fat	≤ 20 g/d
14.	The consumption of drinks with added sugar, such as carbonated drinks should be limited	≤ 20 g/d
15.	Low-fat dairy products should be included in your daily diet	≥ 100 g/d
16.	The consumption of dairy products that contain high levels of saturated fat and/or a high energy content should be limited	≤ 20 g/d
17.	Cooking oils and margarine with a low content of saturated fatty acids and a high content of unsaturated fatty acids should be used in preference to similar products with a great proportion of saturated fatty acids.	Users of cooking oil, liquid margarine or soft margarine and non-users of butter with high content of saturated fatty acids
18.	It is recommended to consume a moderate amount of unsalted nuts (about 140 grams per week)	≥ 20g/d nuts and BMI<25<br>20 g/d ≤ nuts < 30g/d and BMI ≥ 25
19.	Dietary supplements is not recommended	0 unit/d

Table S4: Operationalization of the lifestyle recommendations with a priori cut-off values according to Norwegian food based dietary guidelines included in DIGIKOST dataset [32].

Lifestyle recommendations	Intake/activity required to fulfil the recommendations
1. Consumption of alcohol is not recommended	0 g/d
2. Body mass index (BMI)	Healthy weight (18.5-24.9 kg/m^2)
3. Physical activity	150 min/week of moderate-to vigorous physical activity
4. Tobacco use	0 unit/d

Table S5: Revisions of DIGIKOST-FFQ and DIGIKOST report based on results from focus group interviews and usability testing: Main challenges and issues identified [27] and improvements made.

**DIGIKOST-FFQ**	**Challenges identified**	**Improvements made**
**Completion time**	Time of completion: Got tired at the end, and the questionnaire cannot be longer	Keeping it short and time-effective:<br>- added more introductive questions<br>- removed some items in different categories and aggregated some items to fewer categories
**Layout**	- Challenging in reporting the previous year over several seasons<br>- Challenging to calculate number of slices of bread and use this number when reporting spreads on bread	- Reporting period changed to the last two months<br>- Automatic calculations of bread slices in web-form (front-end function), which is also visible when reporting different spreads used on bread
**Questions**	- Problems to report frequency and portion sizes for some food items<br>- Duration of residence in Norway when you have answered that you were born in Norway.<br>- Problems to report money used on snuff<br>- Missed questions about some specific food items<br>- Missed some specific topics to broaden the understanding of sedentary time<br>- Other missing questions<br>- Missed informative text prior to new food categories and questions on physical activity	- Adjusted frequency and portion sizes for the most challenging food items<br>- Changed questions regarding ethnicity in order to simplify and shorten the questions<br>- Removed questions about money used on snuff<br>- Added questions of some of the missing food items, such as egg, fresh berries as jam, smoothie, other vegetables, legumes<br>- Revised and added more text in sedentary time, such as included transport time and power naps in sedentary question<br>- Added student as an own category for education<br>- Added more text prior to new food categories and physical activity<br>- Added open text-field at the end of the questionnaire allowing the participants to report other items not covered in the web-form
**Pictures**	- Some images were challenging to tell apart different portions or servings<br>- Missed grams as references to the different images visualizing different portions or servings	- Adjusted the specific images to make it easier to tell apart the different portions or servings<br>- Added grams as references to the specific images visualizing different portions or servings
**Motivation**	Individual reports were motivational	Finalized the DIGIKOST report

**DIGIKOST report**	**Challenges identified**	**Improvements made**
	- Challenging to interpret the graphically presentation of the degree of adherence of the Norwegian FBDG with traffic light coloured columns measured in percentage.<br>- Challenging to interpret the adherence to the recommendations presented as health index consisting of 5 lifestyle components, i.e. diet, body fatness (body mass index), physical activity, smoking and alcohol.	- Removed the graphically presentation of adherence.<br>- Removed the presentation of the health index

	- Challenging to interpret the Healthy Eating Plate	- Removed the Healthy Eating Plate

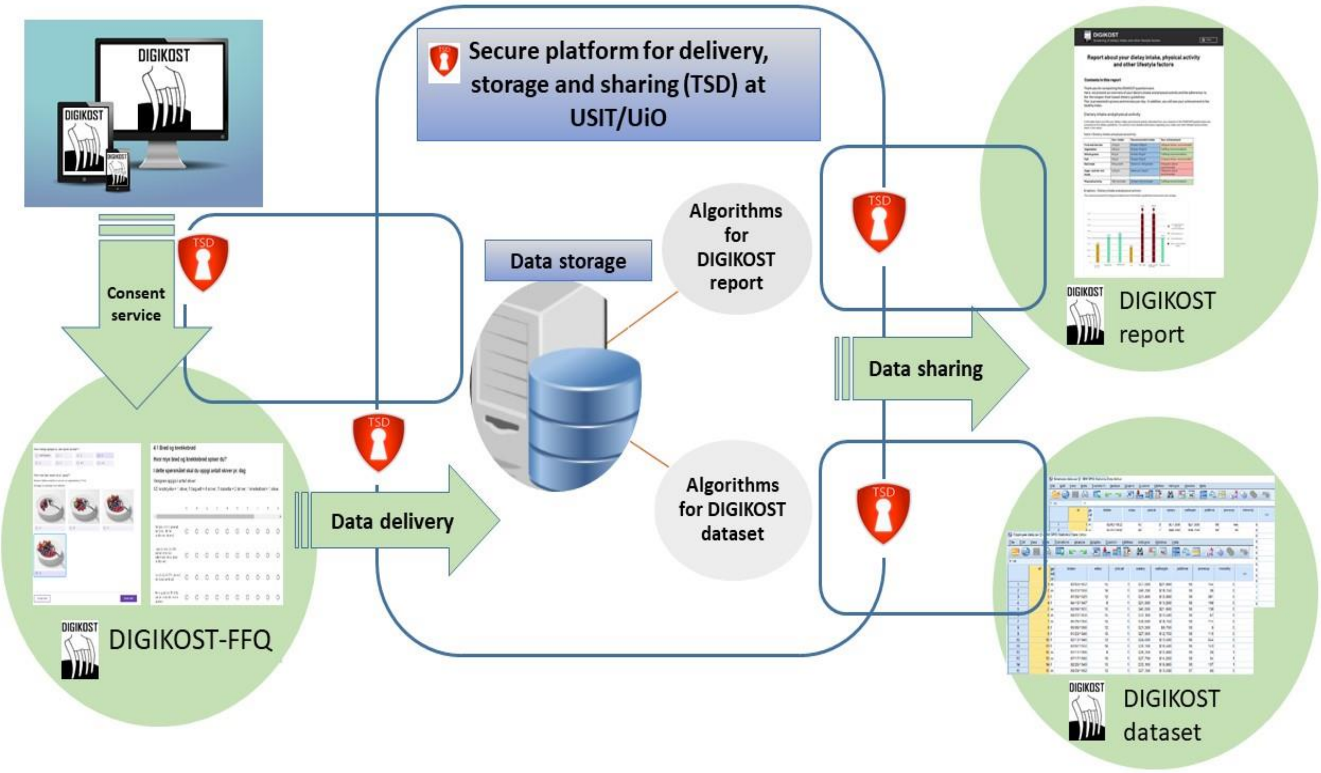

Figure S1**:** Illustration of technical use and data flow in the DIGIKOST application; The DIGIKOST-FFQ provides data on dietary intakes and lifestyle factors, which are delivered to the secure server, TSD. The automatic transformation by the algorithms stored in TSD, generates the DIGIKOST dataset and the DIGIKOST report.

Figure S2: Design view of DIGIKOST-FFQ in printed version (only for demonstration for this paper). The DIGIKOST-FFQ is always used digitally and not in this printed version.

DIGIKOST: Spørreskjema om kosthold, fysisk aktivitet og tobakksvaner

Obligatoriske felter er merket med stjerne *

DIGIKOST

Ditt fødselsnummer *

I denne undersøkelsen spør vi om dine livsstilsvaner, slik som kosthold, fysisk aktivitet og tobakksvaner.

Ha de siste 2 månedene i tankene når du fyller ut spørreskjemaet:

- Vi er klar over at livsstil varierer fra dag til dag, prøv derfor så godt du kan å gi et gjennomsnitt av dine livsstilsvaner.
- Vi er ute etter ditt vanlige inntak av mat og drikke og din aktivitet per uke i løpet av de siste 2 månedene.

Til å hjelpe deg med å bestemme mengder og porsjoner har vi noen steder lagt inn bilder av porsjoner av ulike typer mat.

Bildene er ikke alltid samme matvare som vi spør om, men en som ligner i størrelse og type.

Det vil ta ca. 15 minutter å fylle ut skjemaet.

Samtykke om deltakelse til å fylle ut DIGIKOST spørreskjema *

- ○ Jeg har lest gjennom informasjonen om spørreskjemaet, og er villig til å delta

Sideskift

Obligatoriske felter er merket med stjerne *

1. Frukt og bær

1.1 Epler, pærer eller tilsvarende

Hvor mange ganger pr. uke spiser du epler, pærer eller tilsvarende? *

Her kan du oppgi det du spiser rå, i matlaging og/eller som pålegg.

○ Aldri/Sjelden	○ 1	○ 2	○ 3
○ 4	○ 5	○ 6-7	○ ≥ 8

Hvor mange epler, pærer eller tilsvarende spiser du hver gang? *

Dette elementet vises kun dersom alternativet «5», «6-7», «≥ 8», «1», «2», «3» eller «4» er valgt i spørsmålet «Hvor mange ganger pr. uke spiser du epler, pærer eller tilsvarende?»

Et vanlig eple eller pære veier ca. 135 gram

- 1/2 stk
- 1 stk
- 2 stk
- 3 stk
- ≥ 4 stk

1.2 Appelsin

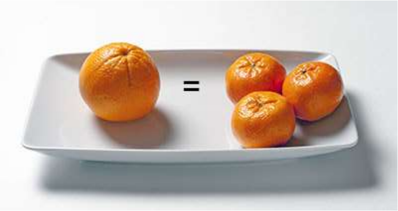

Bildet over viser en vanlig appelsin (195 gram) som tilsvarer ca. 3 små klementiner

Hvor mange ganger pr. uke spiser du appelsiner? *

- Aldri/Sjelden
- 1
- 2
- 3
- 4
- 5
- 6-7
- ≥ 8

Hvor mange vanlige appelsiner spiser du hver gang? *

Dette elementet vises kun dersom alternativet «4», «5», «6-7», «≥ 8», «1», «2» eller «3» er valgt i spørsmålet «Hvor mange ganger pr. uke spiser du appelsiner?»

- 1/2 stk
- 1 stk
- 2 stk
- 3 stk
- ≥ 4 stk

1.3 Banan

Hvor mange ganger pr. uke spiser du banan? *

Her kan du oppgi det du spiser rå, i matlaging og/eller som pålegg.

- Aldri/Sjelden
- 1
- 2
- 3
- 4
- 5
- 6-7
- ≥ 8

Hvor mange bananer spiser du hver gang? *

Dette elementet vises kun dersom alternativet «1», «2», «≥ 8», «3», «4», «5» eller «6-7» er valgt i spørsmålet «Hvor mange ganger pr. uke spiser du banan?»

En vanlig banan veier ca. 120 gram

- 1/2 stk
- 1 stk
- 2 stk
- 3 stk
- ≥ 4 stk

1.4 Liten frukt (f.eks. plommer, druer, kiwi, klementiner)

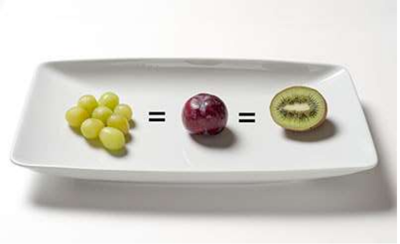

I bildet over er det lik mengde druer (ca. 8 stk), plomme (1 stk) og kiwi (1/2 stk) og som tilsvarer ca. 50 gram.

Hvor mange ganger pr. uke spiser du liten frukt? *

Her kan du oppgi det du spiser rå, i matlaging og/eller som pålegg.

- ◯ Aldri/Sjelden
- ◯ 1
- ◯ 2
- ◯ 3
- ◯ 4
- ◯ 5
- ◯ 6-7
- ◯ ≥ 8

Hvor mange liten frukt spiser du hver gang? *

ⓘ Dette elementet vises kun dersom alternativet «2», «3», «4», «5», «1», «6-7» eller «≥ 8» er valgt i spørsmålet «Hvor mange ganger pr. uke spiser du liten frukt?»

1 liten frukt tilsvarer 1 plomme som vist i bildet ovenfor.

- ◯ 1/2 stk
- ◯ 1 stk
- ◯ 2 stk
- ◯ 3 stk
- ◯ ≥ 4 stk

1.5 Bær (f.eks. jordbær, blåbær, bringebær, tyttebær, kirsebær)

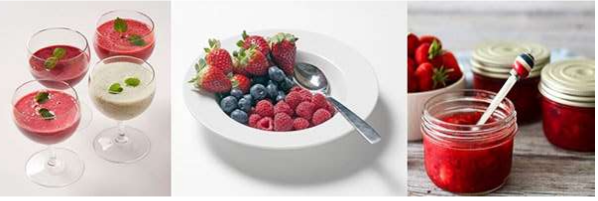

Smoothie inngår også i dette spørsmålet.
Her kan du rapportere frosne og ferske bær, og bær som er rørt ut i litt sukker.

Hvor mange ganger pr. uke spiser du bær? *

- ◯ Aldri/Sjelden
- ◯ 1
- ◯ 2
- ◯ 3
- ◯ 4
- ◯ 5
- ◯ 6-7
- ◯ ≥ 8

Hvor mye bær spiser du hver gang? *

Dette elementet vises kun dersom alternativet «5», «6-7», «≥ 8», «1», «2», «3» eller «4» er valgt i spørsmålet «Hvor mange ganger pr. uke spiser du bær?»

Bærene i bildene nedenfor er servert i en suppetallerken (17cm)

Det ligger en spiseskje i hver tallerken.

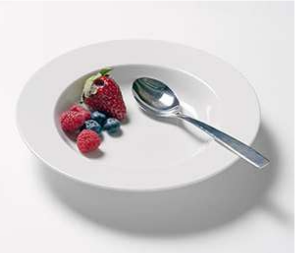

- [ ] A = ca. 50 gram

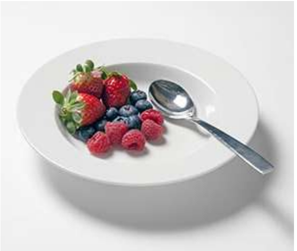

- [ ] B = ca. 100 gram

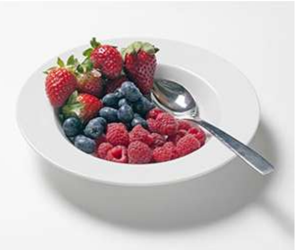

- [ ] C = ca.150 gram

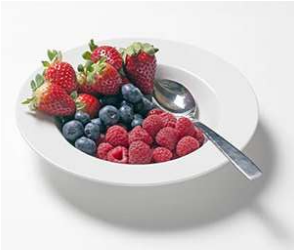

- [ ] D = ca. 250 gram

Sideskift

Obligatoriske felter er merket med stjerne *

2. Nøtter

Inngår nøtter som en del av ditt ukentlige kosthold? *

- [ ] Ja
- [ ] Nei

Dette elementet vises kun dersom alternativet «Ja» er valgt i spørsmålet «Inngår nøtter som en del av ditt ukentlige kosthold?»

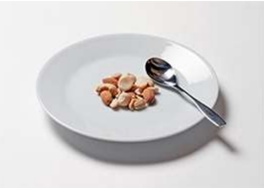

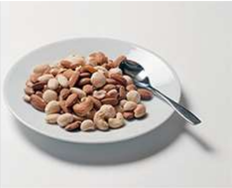

Dette elementet vises kun dersom alternativet «Ja» er valgt i spørsmålet «Inngår nøtter som en del av ditt ukentlige kosthold?»

Bildet til venstre viser ca. 20 gram nøtter (=1 neve)

Bildet til høyre viser ca. 140 gram nøtter (=7 never)

Det ligger en teskje på hver tallerken.

Vi vil først spørre deg om usaltede nøtter og deretter saltede nøtter.

Dette elementet vises kun dersom alternativet «Ja» er valgt i spørsmålet «Inngår nøtter som en del av ditt ukentlige kosthold?»

2.1 Usaltede nøtter (f.eks. mandler, valnøtter, cashewnøtter, ferdige nøtteblandinger, peanøtter)

Hvor mange ganger pr. uke spiser du usaltede nøtter? *

Dette elementet vises kun dersom alternativet «Ja» er valgt i spørsmålet «Inngår nøtter som en del av ditt ukentlige kosthold?»

- Aldri/Sjelden
- 1
- 2
- 3
- 4
- 5
- 6-7
- ≥ 8

Hvor mye usaltede nøtter spiser du hver gang? *

Dette elementet vises kun dersom alternativet «≥ 8», «3», «4», «5», «6-7», «1» eller «2» er valgt i spørsmålet «Hvor mange ganger pr. uke spiser du usaltede nøtter?»

- 1-2 never
- 3-4 never
- 5-6 never
- ≥ 7 never

Dette elementet vises kun dersom alternativet «Ja» er valgt i spørsmålet «Inngår nøtter som en del av ditt ukentlige kosthold?»

Dette elementet vises kun dersom alternativet «Ja» er valgt i spørsmålet «Inngår nøtter som en del av ditt ukentlige kosthold?»

2.2 Saltede nøtter (f.eks. peanøtter, chilinøtter, ferdige nøtteblandinger, pekannøtter, cashewnøtter)

Hvor mange ganger pr. uke spiser du saltede nøtter? *

Dette elementet vises kun dersom alternativet «Ja» er valgt i spørsmålet «Inngår nøtter som en del av ditt ukentlige kosthold?»

- Aldri/Sjelden
- 1
- 2
- 3
- 4
- 5
- 6-7
- ≥ 8

Hvor mye saltede nøtter spiser du hver gang? *

Dette elementet vises kun dersom alternativet «≥ 8», «3», «4», «5», «6-7», «1» eller «2» er valgt i spørsmålet «Hvor mange ganger pr. uke spiser du saltede nøtter?»

- 1-2 never
- 3-4 never
- 5-6 never
- ≥ 7 never

Sideskift

Obligatoriske felter er merket med stjerne *

---

3. Grønnsaker

Vi vil nå spørre deg om de grønnsakene du vanligvis spiser.

Det kan være at du spiser en blanding av det vi spør deg om. Et tips er da å velge litt av hver type grønnsak, så det tilsammen stemmer med det du spiser.

3.1 Gulrot

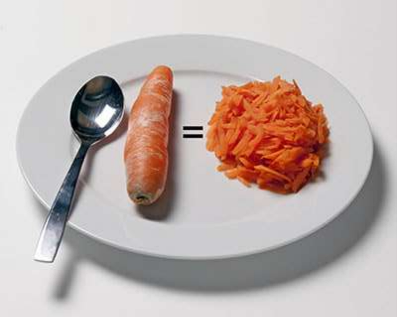

Bildet viser en vanlig gulrot og revet gulrot som tilsvarer 80 gram i mengde.

Gulroten er servert på en middagstallerken (19 cm).

Det ligger en spiseskje på tallerken.

Hvor mange ganger pr. uke spiser du gulrot? *

- ◯ Aldri/Sjelden
- ◯ 1
- ◯ 2
- ◯ 3
- ◯ 4
- ◯ 5
- ◯ 6-7
- ◯ ≥ 8

Hvor mange vanlige gulrøtter spiser du hver gang? *

Dette elementet vises kun dersom alternativet «1», «2», «≥ 8», «3», «4», «5» eller «6-7» er valgt i spørsmålet «Hvor mange ganger pr. uke spiser du gulrot?»

- ◯ 1/2 stk
- ◯ 1 stk
- ◯ 2 stk
- ◯ ≥ 3 stk

3.2 Brokkoli og/eller blomkål

Hvor mange ganger pr. uke spiser du brokkoli og/eller blomkål? *

- ◯ Aldri/Sjelden
- ◯ 1
- ◯ 2
- ◯ 3
- ◯ 4
- ◯ 5
- ◯ 6-7
- ◯ ≥ 8

Hvor mye brokkoli og/eller blomkål spiser du hver gang? *

Dette elementet vises kun dersom alternativet «3», «4», «5», «6-7», «1», «2» eller «≥ 8» er valgt i spørsmålet «Hvor mange ganger pr. uke spiser du brokkoli og/eller blomkål?»

Brokkolien og blomkålen i bildene nedenfor er servert på en middagstallerken (19 cm).

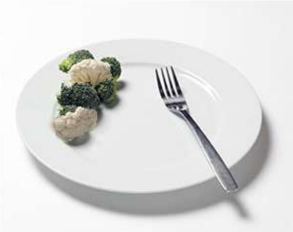

◯ A= ca. 50 gram

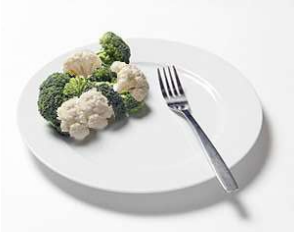

◯ B= ca. 100 gram

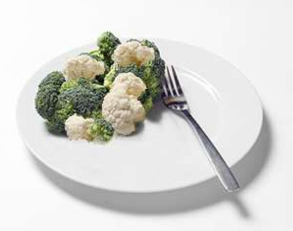

◯ C= ca. 150 gram

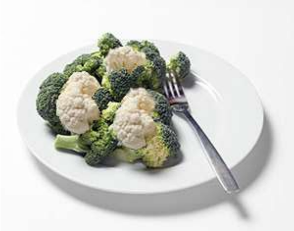

◯ D= ca. 250 gram

3.3 Tomat

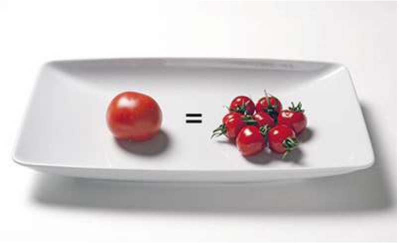

Bildet over viser friske tomater der en vanlig tomat (95 gram) tilsvarer 6-7 små cherrytomater

Hvor mange ganger pr. uke spiser du tomater? *

- Aldri/Sjelden
- 1
- 2
- 3
- 4
- 5
- 6-7
- ≥ 8

Hvor mange vanlige tomater spiser du hver gang? *

Dette elementet vises kun dersom alternativet «≥ 8», «3», «4», «5», «6-7», «1» eller «2» er valgt i spørsmålet «Hvor mange ganger pr. uke spiser du tomater?»

- 1/2 stk
- 1 stk
- 2 stk
- ≥ 3 stk

3.4 Tomatprodukter (f.eks. tomatsaus, hermetiske tomater, ketchup)

Hvor mange ganger pr.uke spiser du tomatprodukter *

- Aldri/Sjelden
- 1
- 2
- 3
- 4
- 5
- 6-7
- ≥ 8

Dette elementet vises kun dersom alternativet «1», «2», «≥ 8», «3», «4», «5» eller «6-7» er valgt i spørsmålet «Hvor mange ganger pr.uke spiser du tomatprodukter»

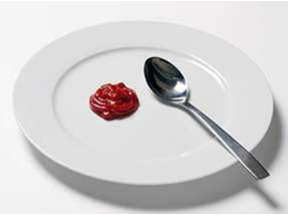

Dette elementet vises kun dersom alternativet «1», «2», «≥ 8», «3», «4», «5» eller «6-7» er valgt i spørsmålet «Hvor mange ganger pr.uke spiser du tomatprodukter»

Bildet over viser mengde tomatprodukt (17 gram) som tilsvarer en spiseskje (ss).

Tomatproduktet i bildet er servert på en middagstallerken (19 cm).

Hvor mye tomatprodukter spiser du hver gang? *

Dette elementet vises kun dersom alternativet «1», «2», «≥ 8», «3», «4», «5» eller «6-7» er valgt i spørsmålet «Hvor mange ganger pr.uke spiser du tomatprodukter»

ss = spiseskje

- 0,5 ss
- 1 ss
- 2 ss
- ≥ 3 ss

3.5 Løk, vårløk og purreløk

Inngår løk, purreløk og/eller vårløk som en del av ditt ukentlige kosthold? *

- Ja
- Nei

Hvor mange ganger pr. uke spiser du løk, vårløk og/eller purreløk? *

Dette elementet vises kun dersom alternativet «Ja» er valgt i spørsmålet «Inngår løk, purreløk og/eller vårløk som en del av ditt ukentlige kosthold?»

- Aldri/Sjelden
- 1
- 2
- 3
- 4
- 5
- 6-7
- ≥ 8

Dette elementet vises kun dersom alternativet «1», «2», «3», «4», «5», «6-7» eller «≥ 8» er valgt i spørsmålet «Hvor mange ganger pr. uke spiser du løk, vårløk og/eller purreløk?»

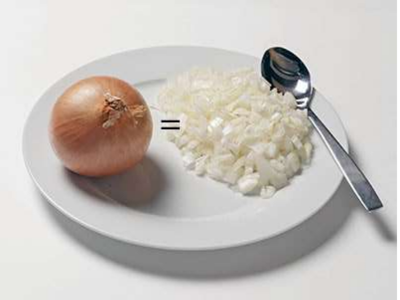

Dette elementet vises kun dersom alternativet «1», «2», «3», «4», «5», «6-7» eller «≥ 8» er valgt i spørsmålet «Hvor mange ganger pr. uke spiser du løk, vårløk og/eller purreløk?»

Bildet viser en vanlig løk og revet løk som tilsvarer 150 gram i mengde.

Løken er servert på en middagstallerken (19 cm).

Det ligger en spiseskje (ss) på tallerken.

Hvor mye hver gang spiser du løk, vårløk og/eller purreløk? *

Dette elementet vises kun dersom alternativet «1», «2», «3», «4», «5», «6-7» eller «≥ 8» er valgt i spørsmålet «Hvor mange ganger pr. uke spiser du løk, vårløk og/eller purreløk?»

1 ss løk = 10 gram

- 1 ss
- 2 ss
- 3 ss
- ≥ 4 ss

3.6 Blandet salat

Hvor mange ganger pr. uke spiser du blandet salat? *

- Aldri/Sjelden
- 1
- 2
- 3
- 4
- 5
- 6-7
- ≥ 8

Hvor mye blandet salat spiser du hver gang? *

Dette elementet vises kun dersom alternativet «5», «6-7», «≥ 8», «1», «2», «3» eller «4» er valgt i spørsmålet «Hvor mange ganger pr. uke spiser du blandet salat?»

Grønnsakene i bildene nedenfor er servert på en middagstallerken (19 cm).

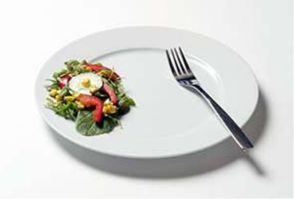

A = ca. 25 gram

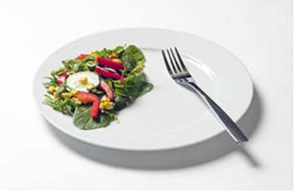

B = ca. 50 gram

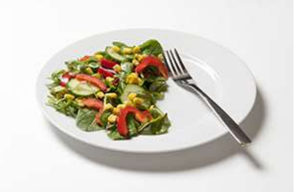

C = ca. 100 gram

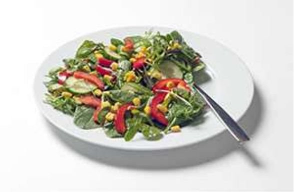

D = ca. 150 gram

3.7 Rotgrønnsaker (f.eks. kålrot, sellerirot, persillerot, reddik)

Hvor mange ganger pr. uke spiser du rotgrønnsaker? *

- Aldri/Sjelden
- 1
- 2
- 3
- 4
- 5
- 6-7
- ≥ 8

Hvor mye rotgrønnsaker spiser du hver gang? *

Dette elementet vises kun dersom alternativet «5», «6-7», «≥ 8», «1», «2», «3» eller «4» er valgt i spørsmålet «Hvor mange ganger pr. uke spiser du rotgrønnsaker?»

Grønnsakene i bildene nedenfor er servert på en middagstallerken (19 cm).

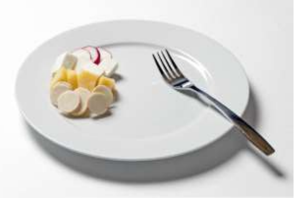

A = ca. 50 gram

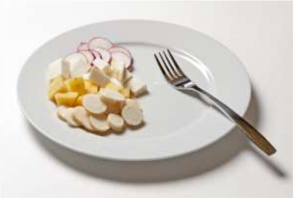

B = ca. 100 gram

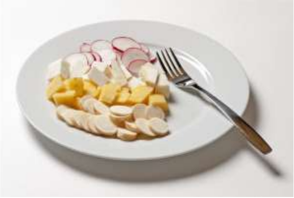

C = ca. 150 gram

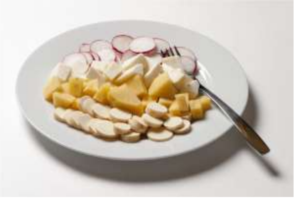

D = ca. 250 gram

3.8 Belgfrukter (f.eks. bønner, erter, linser, kikerter)

Hvor mange ganger pr. uke spiser du belgfrukter? *

- ◯ Aldri/Sjelden
- ◯ 1
- ◯ 2
- ◯ 3
- ◯ 4
- ◯ 5
- ◯ 6-7
- ◯ ≥ 8

Hvor mye belgfrukter spiser du hver gang? *

> Dette elementet vises kun dersom alternativet «2», «3», «1», «6-7», «≥ 8», «4» eller «5» er valgt i spørsmålet «Hvor mange ganger pr. uke spiser du belgfrukter?»

- ◯ 0,5 dl
- ◯ 1 dl
- ◯ 2 dl
- ◯ ≥ 3 dl

3.9 Andre grønnsaker (f.eks. avokado, aubergin, squash)

Hvor mange ganger pr. uke spiser du andre grønnsaker ? *

- ◯ Aldri/Sjelden
- ◯ 1
- ◯ 2
- ◯ 3
- ◯ 4
- ◯ 5
- ◯ 6-7
- ◯ ≥ 8

Hvor mye andre grønnsaker spiser du hver gang? *

> Dette elementet vises kun dersom alternativet «1», «2», «5», «6-7», «3», «4» eller «≥ 8» er valgt i spørsmålet «Hvor mange ganger pr. uke spiser du andre grønnsaker ?»

En stor avokado, en vanlig aubergin og en vanlig squash veier hver seg omtrent 250 gram.

- ◯ 50 gram
- ◯ 100 gram
- ◯ 150 gram
- ◯ ≥ 250 gram

Sideskift

Obligatoriske felter er merket med stjerne *

---

4. Brød, knekkebrød og pålegg

Brødskalaens fire kategorier har vi satt inn for å hjelpe deg å vurdere grovheten på brødproduktene du spiser.

0-25% sammalt mel/hele korn

25-50% sammalt mel/hele korn

50-75% sammalt mel/hele korn

75-100% sammalt mel/hele korn

4.1 Brød og knekkebrød

Hvor mye brød og knekkebrød spiser du?

I dette spørsmålet skal du oppgi antall skiver pr. dag

Mengden oppgis i antall skiver:

1/2 rundstykke = 1 skive; 1 baguett = 4 skiver; 1 ciabatta = 2 skiver; 1 knekkebrød= 1 skive

	0	1	2	3	4	5	6	7	8	9
Fint brød, 0-25% sammalt mel (f.eks. loff, fine rundstykker, ciabatta)	○	○	○	○	○	○	○	○	○	○
Halvgrovt brød, 25-50% sammalt mel (f.eks. helkornbrød, kneip, grove rundstykker)	○	○	○	○	○	○	○	○	○	○
Grovt brød, 50-75% sammalt mel (f.eks.havrebrød)	○	○	○	○	○	○	○	○	○	○
Ekstra grovbrød, 75-100% sammalt mel (f.eks. mørkt rugbrød)	○	○	○	○	○	○	○	○	○	○
Fint knekkebrød (f.eks. kavring, frokost knekkebrød)	○	○	○	○	○	○	○	○	○	○
Grovt knekkebrød (f.eks. Husman, Sport, Solruta)	○	○	○	○	○	○	○	○	○	○

Vi har regnet ut at du bruker 0 brødskiver og knekkebrød per uke

4.2 Pålegg

Man kan bruke flere pålegg pr. brødskive eller knekkebrød

Hvor mye pålegg har du vanligvis på de 0 brødskivene og eller knekkebrødene (viser til antall brødskiver og knekkebrød per uke beregnet i spørsmålet ovenfor)?

	0	1-3 skiver	4-7 skiver	8-12 skiver	13-18 skiver	19-25 skiver	≥ 26 skiver
Fete oster som pålegg (f.eks. helfet Norvegia, helfet Jarlsberg, brunost, prim, brie) *	○	○	○	○	○	○	○
Magre oster som pålegg (f.eks. lett Norvegia, lett Jarlsberg, cottage cheese) *	○	○	○	○	○	○	○
Fiskepålegg (f.eks. makrell i tomat, røket/gravet laks, sild) *	○	○	○	○	○	○	○
Rødt kjøtt (f.eks. salami, skinke, servelat, leverpostei) *	○	○	○	○	○	○	○
Hvitt kjøtt (f.eks. kyllingpålegg, kalkunpålegg, kyllingleverpostei) *	○	○	○	○	○	○	○
Pålegg med sukker (f.eks. honning, syltetøy, nøttepålegg) *	○	○	○	○	○	○	○
Egg (kokt, stekt, eggerøre) *	○	○	○	○	○	○	○

Sideskift

Obligatoriske felter er merket med stjerne *

4.3 Grøt og kornblandinger

Inngår grøt og/eller kornblandinger som en del av ditt ukentlige kosthold? *

- Ja
- Nei

Dette elementet vises kun dersom alternativet «Ja» er valgt i spørsmålet «Inngår grøt og/eller kornblandinger som en del av ditt ukentlige kosthold?»

Dette elementet vises kun dersom alternativet «Ja» er valgt i spørsmålet «Inngår grøt og/eller kornblandinger som en del av ditt ukentlige kosthold?»

4.3.1 Havregrøt/ byggrynsgrøt

Hvor mange ganger pr. uke spiser du havregrøt/ byggrynsgrøt? *

Dette elementet vises kun dersom alternativet «Ja» er valgt i spørsmålet «Inngår grøt og/eller kornblandinger som en del av ditt ukentlige kosthold?»

- Aldri/Sjelden
- 1
- 2
- 3
- 4
- 5
- 6-7
- ≥ 8

Hvor mye havregrøt/ byggrynsgrøt spiser du hver gang? *

Dette elementet vises kun dersom alternativet «5», «6-7», «≥ 8», «1», «2», «3» eller «4» er valgt i spørsmålet «Hvor mange ganger pr. uke spiser du havregrøt/ byggrynsgrøt?»

Grøten i bildene nedenfor er servert i en suppetallerken (17 cm).

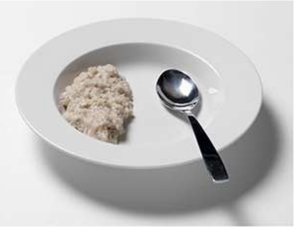

- A = ca. 90 gram

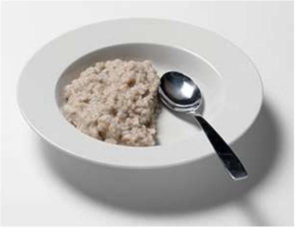

- B = ca. 180 gram

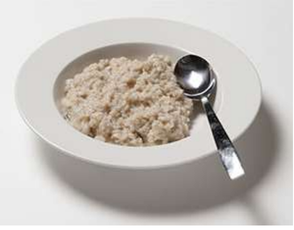

- C = ca. 270 gram

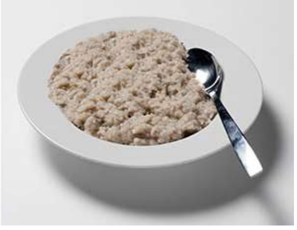

- D = ca. 360 gram

Dette elementet vises kun dersom alternativet «Ja» er valgt i spørsmålet «Inngår grøt og/eller kornblandinger som en del av ditt ukentlige kosthold?»

Dette elementet vises kun dersom alternativet «Ja» er valgt i spørsmålet «Inngår grøt og/eller kornblandinger som en del av ditt ukentlige kosthold?»

4.3.2 Risengrynsgrøt

Hvor mange ganger pr. uke spiser du risengrynsgrøt? *

Dette elementet vises kun dersom alternativet «Ja» er valgt i spørsmålet «Inngår grøt og/eller kornblandinger som en del av ditt ukentlige kosthold?»

- ○ Aldri/Sjelden
- ○ 1
- ○ 2
- ○ 3
- ○ 4
- ○ 5
- ○ 6-7
- ○ ≥ 8

Hvor mye risengrynsgrøt spiser du hver gang? *

Dette elementet vises kun dersom alternativet «1», «2», «3», «4», «5», «6-7» eller «≥ 8» er valgt i spørsmålet «Hvor mange ganger pr. uke spiser du risengrynsgrøt?»

Grøten i bildene nedenfor er servert i en suppetallerken (17 cm)

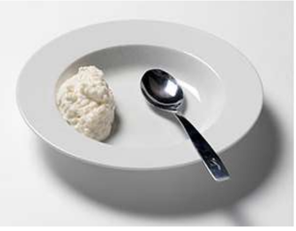

○ A = ca. 90 gram

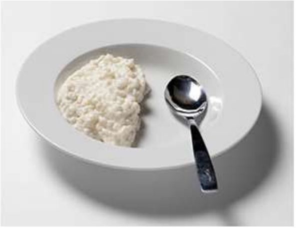

○ B = ca. 180 gram

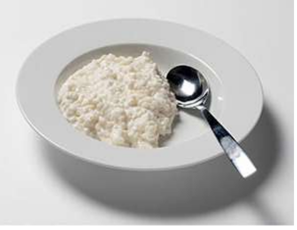

○ C = ca. 270 gram

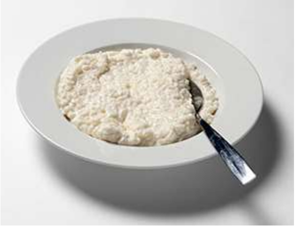

○ D = ca. 360 gram

Dette elementet vises kun dersom alternativet «Ja» er valgt i spørsmålet «Inngår grøt og/eller kornblandinger som en del av ditt ukentlige kosthold?»

Dette elementet vises kun dersom alternativet «Ja» er valgt i spørsmålet «Inngår grøt og/eller kornblandinger som en del av ditt ukentlige kosthold?»

4.3.3 Usøtede kornblandinger (f.eks. 4-korn, havregryn, musli)

Hvor mange ganger pr. uke spiser du usøtede kornblandinger? *

Dette elementet vises kun dersom alternativet «Ja» er valgt i spørsmålet «Inngår grøt og/eller kornblandinger som en del av ditt ukentlige kosthold?»

- ○ Aldri/Sjelden
- ○ 1
- ○ 2
- ○ 3
- ○ 4
- ○ 5
- ○ 6-7
- ○ ≥ 8

Hvor mye usøtet kornblanding spiser du hver gang? *

Dette elementet vises kun dersom alternativet «5», «6-7», «≥ 8», «1», «2», «3» eller «4» er valgt i spørsmålet «Hvor mange ganger pr. uke spiser du usøtede kornblandinger?»

Frokostblandingen i bildene nedenfor er servert i en suppetallerken (17 cm)

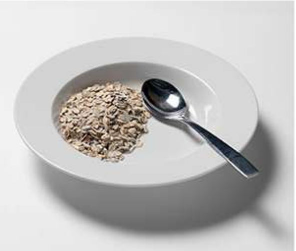

○ A = ca. 40 gram

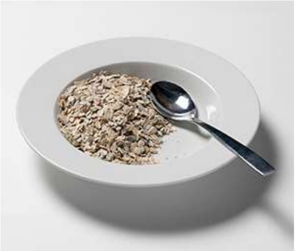

○ B = ca. 80 gram

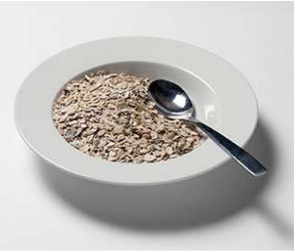

○ C = ca. 120 gram

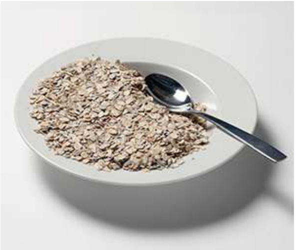

○ D = ca. 160 gram

Dette elementet vises kun dersom alternativet «Ja» er valgt i spørsmålet «Inngår grøt og/eller kornblandinger som en del av ditt ukentlige kosthold?»

Dette elementet vises kun dersom alternativet «Ja» er valgt i spørsmålet «Inngår grøt og/eller kornblandinger som en del av ditt ukentlige kosthold?»

4.3.4 Søtede kornblandinger (f.eks. Corn flakes, Chocofrokost)

Hvor mange ganger pr. uke spiser du søtede kornblandinger? *

Dette elementet vises kun dersom alternativet «Ja» er valgt i spørsmålet «Inngår grøt og/eller kornblandinger som en del av ditt ukentlige kosthold?»

- ◯ Aldri/Sjelden
- ◯ 1
- ◯ 2
- ◯ 3
- ◯ 4
- ◯ 5
- ◯ 6-7
- ◯ ≥ 8

Hvor mye søtet kornblanding spiser du hver gang? *

Dette elementet vises kun dersom alternativet «5», «6-7», «≥ 8», «1», «2», «3» eller «4» er valgt i spørsmålet «Hvor mange ganger pr. uke spiser du søtede kornblandinger?»

Den søtede kornblandingen i bildene nedenfor er servert i en suppetallerken (17 cm)

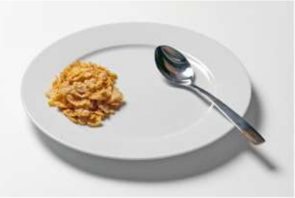

◯ A = ca. 14 gram

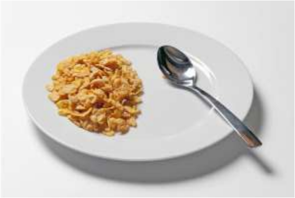

◯ B = ca. 28 gram

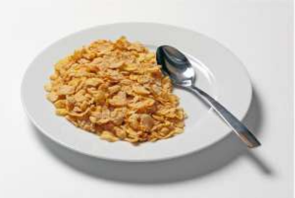

◯ C = ca. 42 gram

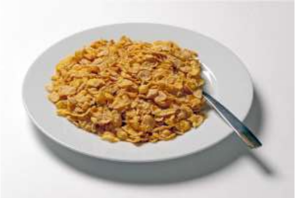

◯ D = ca. 56 gram

Sideskift

Obligatoriske felter er merket med stjerne *

5. Margarin, smør og olje

5.1 Bruker du vanligvis margarin, smør eller olje på brød, baguette og/eller rundstykker? *

- ◯ Ja
- ◯ Nei, vanligvis ikke

Hva bruker du oftest på brød, baguette eller rundstykker? *

ℹ Dette elementet vises kun dersom alternativet «Ja» er valgt i spørsmålet «5.1 Bruker du vanligvis margarin, smør eller olje på brød, baguette og/eller rundstykker?»

- ◯ Margarin (f.eks. Soft Flora, Vita, Soft Oliven)
- ◯ Smør (f.eks. Bremykt, meierismør)
- ◯ Oljer (f.eks. olivenolje, soyaolje, rapsolje, Vita hjertego)

5.2 Bruker du vanligvis margarin, smør og olje til matlaging? *

- ◯ Ja
- ◯ Nei, vanligvis ikke

Hva bruker du oftest til matlaging? *

ℹ Dette elementet vises kun dersom alternativet «Ja» er valgt i spørsmålet «5.2 Bruker du vanligvis margarin, smør og olje til matlaging?»

- ◯ Margarin (f.eks. Soft Flora, Vita, Soft Oliven)
- ◯ Smør (f.eks. Bremykt, meierismør)
- ◯ Oljer (f.eks. olivenolje, soyaolje, rapsolje, Vita hjertego)

Sideskift

Obligatoriske felter er merket med stjerne *

---

6. Fisk

Inngår fisk som en del av ditt ukentlige kosthold? *

- ◯ Ja
- ◯ Nei

ℹ Dette elementet vises kun dersom alternativet «Ja» er valgt i spørsmålet «Inngår fisk som en del av ditt ukentlige kosthold?»

Vi vil først spørre om fet fisk, deretter mager fisk og bearbeidet fisk.

Dette elementet vises kun dersom alternativet «Ja» er valgt i spørsmålet «Inngår fisk som en del av ditt ukentlige kosthold?»

6.1 Fet fisk (f.eks. laks, ørret, sild, kveite)

Hvor mange ganger pr. uke spiser du fet fisk? *

Dette elementet vises kun dersom alternativet «Ja» er valgt i spørsmålet «Inngår fisk som en del av ditt ukentlige kosthold?»

- ◯ Aldri/Sjelden
- ◯ 1
- ◯ 2
- ◯ 3
- ◯ 4
- ◯ 5
- ◯ 6-7
- ◯ ≥ 8

Hvor mye fet fisk spiser du hver gang? *

Dette elementet vises kun dersom alternativet «1», «2», «3», «4», «5», «6-7» eller «≥ 8» er valgt i spørsmålet «Hvor mange ganger pr. uke spiser du fet fisk?»

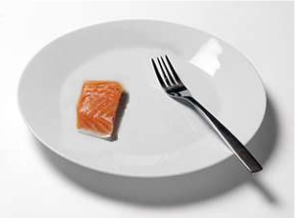

◯ A: 0,5 porsjon (ca. 62 gram)

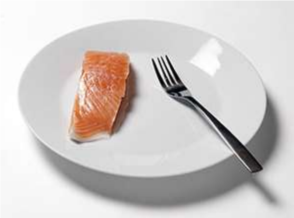

◯ B: 1 porsjon (ca. 125 gram)

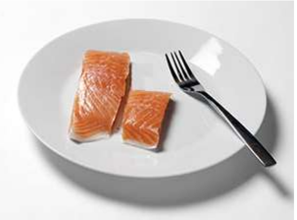

◯ C: 1,5 porsjoner (ca. 187 gram)

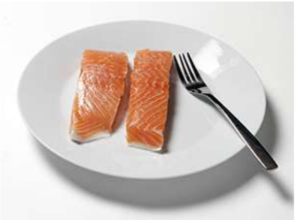

◯ D: 2 porsjoner (ca. 250 gram) eller mer

Dette elementet vises kun dersom alternativet «Ja» er valgt i spørsmålet «Inngår fisk som en del av ditt ukentlige kosthold?»

Dette elementet vises kun dersom alternativet «Ja» er valgt i spørsmålet «Inngår fisk som en del av ditt ukentlige kosthold?»

6.2 Mager fisk (f.eks. torsk, sei, hyse, rødspette, breiflabb)

Hvor mange ganger pr. uke spiser du mager fisk? *

Dette elementet vises kun dersom alternativet «Ja» er valgt i spørsmålet «Inngår fisk som en del av ditt ukentlige kosthold?»

- Aldri/Sjelden
- 1
- 2
- 3
- 4
- 5
- 6-7
- ≥ 8

Hvor mye mager fisk spiser du hver gang? *

Dette elementet vises kun dersom alternativet «3», «4», «5», «6-7», «1», «2» eller «≥ 8» er valgt i spørsmålet «Hvor mange ganger pr. uke spiser du mager fisk?»

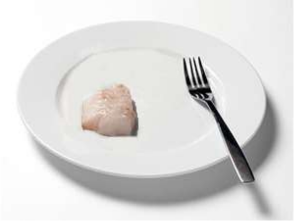

A: 0,5 porsjon (62 gram)

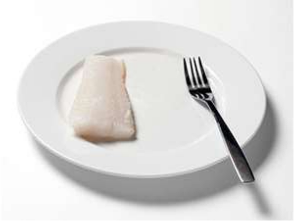

B: 1 porsjon (ca. 125 gram)

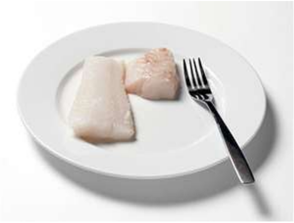

C: 1,5 porsjoner (ca. 187 gram)

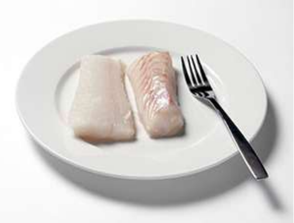

D: 2 porsjoner (ca. 250 gram) eller mer

Dette elementet vises kun dersom alternativet «Ja» er valgt i spørsmålet «Inngår fisk som en del av ditt ukentlige kosthold?»

Dette elementet vises kun dersom alternativet «Ja» er valgt i spørsmålet «Inngår fisk som en del av ditt ukentlige kosthold?»

6.3 Bearbeidet fisk (f.eks. fiskekaker, fiskegrateng, fiskeboller, fiskepudding)

Hvor mange ganger pr. uke spiser du bearbeidet fisk? *

Dette elementet vises kun dersom alternativet «Ja» er valgt i spørsmålet «Inngår fisk som en del av ditt ukentlige kosthold?»

- Aldri/Sjelden
- 1
- 2
- 3
- 4
- 5
- 6-7
- ≥ 8

Dette elementet vises kun dersom alternativet «5», «6-7», «≥ 8», «1», «2», «3» eller «4» er valgt i spørsmålet «Hvor mange ganger pr. uke spiser du bearbeidet fisk?»

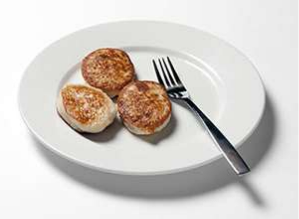

Dette elementet vises kun dersom alternativet «5», «6-7», «≥ 8», «1», «2», «3» eller «4» er valgt i spørsmålet «Hvor mange ganger pr. uke spiser du bearbeidet fisk?»

Bildet over viser 1 porsjon med mager fisk, tilsvarende 150 gram.

Hvor mye bearbeidet fisk spiser du hver gang? *

Dette elementet vises kun dersom alternativet «5», «6-7», «≥ 8», «1», «2», «3» eller «4» er valgt i spørsmålet «Hvor mange ganger pr. uke spiser du bearbeidet fisk?»

- 0,25 porsjon
- 0,5 porsjon
- 1 porsjon
- 1,5 porsjoner
- 2 porsjoner
- ≥ 3 porsjoner

Sideskift

Obligatoriske felter er merket med stjerne *

7. Kjøtt

Inngår kjøtt som en del av ditt ukentlige kosthold? *

- Ja
- Nei

Vi vil først spørre deg om rødt kjøtt og deretter hvitt kjøtt.

Dette elementet vises kun dersom alternativet «Ja» er valgt i spørsmålet «Inngår kjøtt som en del av ditt ukentlige kosthold?»

7.1 Rødt kjøtt (f.eks. storfe, svin eller sau/lam)

Dette elementet vises kun dersom alternativet «Ja» er valgt i spørsmålet «Inngår kjøtt som en del av ditt ukentlige kosthold?»

7.1.1 Bearbeidet rødt kjøtt (f.eks. pølser, hamburger, kjøttboller, sommer-kotelett, kjøttdeig)

Dette elementet vises kun dersom alternativet «Ja» er valgt i spørsmålet «Inngår kjøtt som en del av ditt ukentlige kosthold?»

Bearbeidet kjøtt er røkt, saltet eller konservert med nitrat eller nitritt

Hvor mange ganger pr. uke spiser du bearbeidet rødt kjøtt? *

Dette elementet vises kun dersom alternativet «Ja» er valgt i spørsmålet «Inngår kjøtt som en del av ditt ukentlige kosthold?»

- ◯ Aldri/Sjelden
- ◯ 1
- ◯ 2
- ◯ 3
- ◯ 4
- ◯ 5
- ◯ 6-7
- ◯ ≥ 8

Dette elementet vises kun dersom alternativet «5», «6-7», «≥ 8», «1», «2», «3» eller «4» er valgt i spørsmålet «Hvor mange ganger pr. uke spiser du bearbeidet rødt kjøtt?»

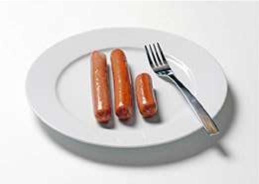
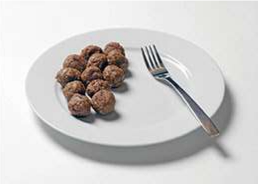
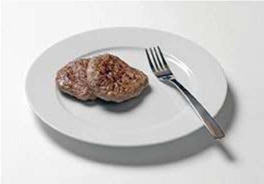

Dette elementet vises kun dersom alternativet «5», «6-7», «≥ 8», «1», «2», «3» eller «4» er valgt i spørsmålet «Hvor mange ganger pr. uke spiser du bearbeidet rødt kjøtt?»

Bildet over viser 1 porsjon bearbeidet rødt kjøtt (150 g). Det er lik mengde pølser, kjøttboller og karbonader

Hvor mye bearbeidet rødt kjøtt spiser du hver gang? *

Dette elementet vises kun dersom alternativet «5», «6-7», «≥ 8», «1», «2», «3» eller «4» er valgt i spørsmålet «Hvor mange ganger pr. uke spiser du bearbeidet rødt kjøtt?»

- ◯ 0,25 porsjon
- ◯ 0,5 porsjon
- ◯ 1 porsjon
- ◯ 1,5 porsjoner
- ◯ 2 porsjoner
- ◯ ≥ 3 porsjoner

Dette elementet vises kun dersom alternativet «Ja» er valgt i spørsmålet «Inngår kjøtt som en del av ditt ukentlige kosthold?»

Dette elementet vises kun dersom alternativet «Ja» er valgt i spørsmålet «Inngår kjøtt som en del av ditt ukentlige kosthold?»

7.1.2 Rødt kjøtt, ikke bearbeidet (f.eks. biff, stek, grytekjøtt)

Hvor mange ganger pr. uke spiser du rødt kjøtt, ikke bearbeidet? *

Dette elementet vises kun dersom alternativet «Ja» er valgt i spørsmålet «Inngår kjøtt som en del av ditt ukentlige kosthold?»

- ◯ Aldri/Sjelden
- ◯ 1
- ◯ 2
- ◯ 3
- ◯ 4
- ◯ 5
- ◯ 6-7
- ◯ ≥ 8

Dette elementet vises kun dersom alternativet «≥ 8», «3», «4», «5», «6-7», «1» eller «2» er valgt i spørsmålet «Hvor mange ganger pr. uke spiser du rødt kjøtt, ikke bearbeidet?»

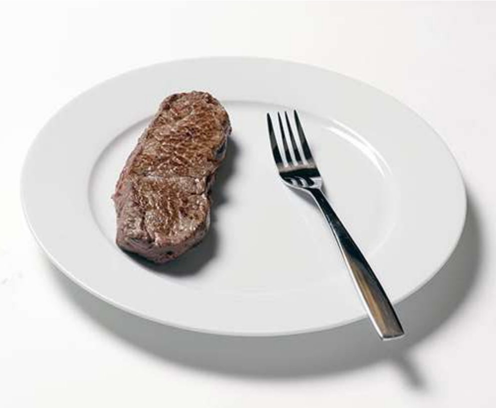

Dette elementet vises kun dersom alternativet «≥ 8», «3», «4», «5», «6-7», «1» eller «2» er valgt i spørsmålet «Hvor mange ganger pr. uke spiser du rødt kjøtt, ikke bearbeidet?»

Dette bilde viser 1 porsjon rødt kjøtt, ikke bearbeidet, tilsvarende 150 gram og som er servert på en middagstallerken (19 cm).

Hvor mye rødt kjøtt, ikke bearbeidet, spiser du hver gang? *

Dette elementet vises kun dersom alternativet «≥ 8», «3», «4», «5», «6-7», «1» eller «2» er valgt i spørsmålet «Hvor mange ganger pr. uke spiser du rødt kjøtt, ikke bearbeidet?»

- ○ 0,25 porsjon
- ○ 0,5 porsjon
- ○ 1 porsjon
- ○ 1,5 porsjoner
- ○ 2 porsjoner
- ○ ≥ 3 porsjoner

Sideskift

Obligatoriske felter er merket med stjerne *

Dette elementet vises kun dersom alternativet «Ja» er valgt i spørsmålet «Inngår kjøtt som en del av ditt ukentlige kosthold?»

7.2 Hvitt kjøtt (f.eks. kylling, kalkun)

Dette elementet vises kun dersom alternativet «Ja» er valgt i spørsmålet «Inngår kjøtt som en del av ditt ukentlige kosthold?»

7.2.1 Bearbeidet hvitt kjøtt (f.eks. pølser, kjøttboller, hamburger)

Hvor mange ganger pr. uke spiser du bearbeidet hvitt kjøtt? *

Dette elementet vises kun dersom alternativet «Ja» er valgt i spørsmålet «Inngår kjøtt som en del av ditt ukentlige kosthold?»

- ○ Aldri/Sjelden
- ○ 1
- ○ 2
- ○ 3
- ○ 4
- ○ 5
- ○ 6-7
- ○ ≥ 8

Dette elementet vises kun dersom alternativet «5», «6-7», «≥ 8», «1», «2», «3» eller «4» er valgt i spørsmålet «Hvor mange ganger pr. uke spiser du bearbeidet hvitt kjøtt?»

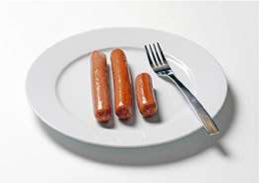 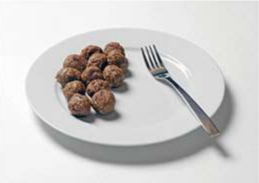 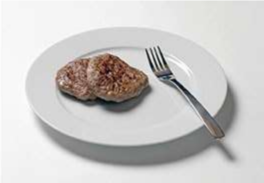

Dette elementet vises kun dersom alternativet «5», «6-7», «≥ 8», «1», «2», «3» eller «4» er valgt i spørsmålet «Hvor mange ganger pr. uke spiser du bearbeidet hvitt kjøtt?»

Bildet over viser 1 porsjon bearbeidet hvitt kjøtt (150 g). Det er lik mengde pølser, kjøttboller og karbonader.

Hvor mye bearbeidet hvitt kjøtt spiser du hver gang? *

Dette elementet vises kun dersom alternativet «5», «6-7», «≥ 8», «1», «2», «3» eller «4» er valgt i spørsmålet «Hvor mange ganger pr. uke spiser du bearbeidet hvitt kjøtt?»

- ○ 0,25 porsjon
- ○ 0,5 porsjon
- ○ 1 porsjon
- ○ 1,5 porsjoner
- ○ 2 porsjoner
- ○ ≥ 3 porsjoner

Dette elementet vises kun dersom alternativet «5», «6-7», «≥ 8», «1», «2», «3» eller «4» er valgt i spørsmålet «Hvor mange ganger pr. uke spiser du bearbeidet hvitt kjøtt?»

Dette elementet vises kun dersom alternativet «Ja» er valgt i spørsmålet «Inngår kjøtt som en del av ditt ukentlige kosthold?»

7.2.2 Hvitt kjøtt, ikke bearbeidet (f.eks. kyllingfilet, kalkunbrystfilet)

Hvor mange ganger pr. uke spiser du hvitt kjøtt, ikke bearbeidet? *

Dette elementet vises kun dersom alternativet «Ja» er valgt i spørsmålet «Inngår kjøtt som en del av ditt ukentlige kosthold?»

- ○ Aldri/Sjelden
- ○ 1
- ○ 2
- ○ 3
- ○ 4
- ○ 5
- ○ 6-7
- ○ ≥ 8

Dette elementet vises kun dersom alternativet «≥ 8», «3», «4», «5», «6-7», «1» eller «2» er valgt i spørsmålet «Hvor mange ganger pr. uke spiser du hvitt kjøtt, ikke bearbeidet?»

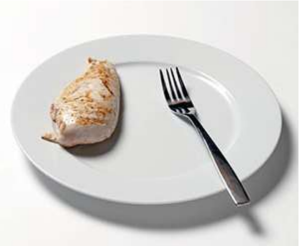

Dette elementet vises kun dersom alternativet «≥ 8», «3», «4», «5», «6-7», «1» eller «2» er valgt i spørsmålet «Hvor mange ganger pr. uke spiser du hvitt kjøtt, ikke bearbeidet?»

Dette bildet viser en kyllingfilet som tilsvarer 1 porsjon hvitt kjøtt (150 g) og som er servert på en middagstallerken (19 cm).

Hvor mye hvitt kjøtt, ikke bearbeidet, spiser du hver gang? *

Dette elementet vises kun dersom alternativet «≥ 8», «3», «4», «5», «6-7», «1» eller «2» er valgt i spørsmålet «Hvor mange ganger pr. uke spiser du hvitt kjøtt, ikke bearbeidet?»

- 0,25 porsjon
- 0,5 porsjon
- 1 porsjon
- 1,5 porsjoner
- 2 porsjoner
- ≥ 3 porsjoner

Sideskift

Obligatoriske felter er merket med stjerne *

8.0 Yoghurt, rømme, creme fraiche eller liknende

Inngår yoghurt, rømme, creme fraiche eller liknende som en del av ditt ukentlige kosthold? *

- Ja
- Nei

Dette elementet vises kun dersom alternativet «Ja» er valgt i spørsmålet «Inngår yoghurt, rømme, creme fraiche eller liknende som en del av ditt ukentlige kosthold?»

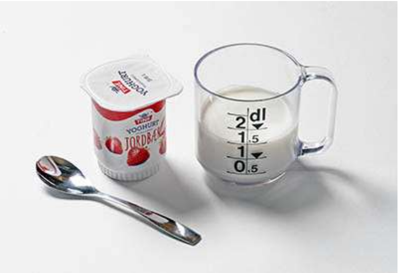

Dette elementet vises kun dersom alternativet «Ja» er valgt i spørsmålet «Inngår yoghurt, rømme, creme fraiche eller liknende som en del av ditt ukentlige kosthold?»

Bildet viser 1,5 dl yoghurt som tilsvarer ett lite beger med yoghurt. Det ligger en teskje ved siden av yoghurten.

Dette elementet vises kun dersom alternativet «Ja» er valgt i spørsmålet «Inngår yoghurt, rømme, creme fraiche eller liknende som en del av ditt ukentlige kosthold?»

8.1 Lett yoghurt (f.eks. all youghurt med "lett", "0%", "0,1%" i navnet eller yoghurt naturell)

Hvor mange ganger pr. uke spiser du lett yoghurt? *

Dette elementet vises kun dersom alternativet «Ja» er valgt i spørsmålet «Inngår yoghurt, rømme, creme fraiche eller liknende som en del av ditt ukentlige kosthold?»

- Aldri/Sjelden
- 1
- 2
- 3
- 4
- 5
- 6-7
- ≥ 8

Hvor mye lett yoghurt spiser du hver gang? *

Dette elementet vises kun dersom alternativet «≥ 8», «3», «4», «5», «6-7», «1» eller «2» er valgt i spørsmålet «Hvor mange ganger pr. uke spiser du lett yoghurt?»

- 0,5 dl
- 1 dl
- 1,5 dl
- 2 dl
- 2,5 dl
- ≥ 3 dl

Dette elementet vises kun dersom alternativet «Ja» er valgt i spørsmålet «Inngår yoghurt, rømme, creme fraiche eller liknende som en del av ditt ukentlige kosthold?»

Dette elementet vises kun dersom alternativet «Ja» er valgt i spørsmålet «Inngår yoghurt, rømme, creme fraiche eller liknende som en del av ditt ukentlige kosthold?»

8.2 Yoghurt (f.eks. God morgen yoghurt, fruktyoghurt)

Hvor mange ganger pr. uke spiser du yoghurt? *

Dette elementet vises kun dersom alternativet «Ja» er valgt i spørsmålet «Inngår yoghurt, rømme, creme fraiche eller liknende som en del av ditt ukentlige kosthold?»

- Aldri/Sjelden
- 1
- 2
- 3
- 4
- 5
- 6-7
- ≥ 8

Hvor mye yoghurt spiser du hver gang? *

Dette elementet vises kun dersom alternativet «5», «6-7», «≥ 8», «1», «2», «3» eller «4» er valgt i spørsmålet «Hvor mange ganger pr. uke spiser du yoghurt?»

- 0,5 dl
- 1 dl
- 1,5 dl
- 2 dl
- 2,5 dl
- ≥ 3 dl

Dette elementet vises kun dersom alternativet «Ja» er valgt i spørsmålet «Inngår yoghurt, rømme, creme fraiche eller liknende som en del av ditt ukentlige kosthold?»

9. Rømme, creme fraiche og liknende

Dette elementet vises kun dersom alternativet «Ja» er valgt i spørsmålet «Inngår yoghurt, rømme, creme fraiche eller liknende som en del av ditt ukentlige kosthold?»

9.1 Rømme, creme fraiche o.l. med lavt fettinnhold (f.eks. lettrømme, mager kesam, eller inneholder mindre enn 20% fett)

Hvor mange ganger pr. uke spiser du rømme, creme fraiche o.l. med lavt fettinnhold? *

Dette elementet vises kun dersom alternativet «Ja» er valgt i spørsmålet «Inngår yoghurt, rømme, creme fraiche eller liknende som en del av ditt ukentlige kosthold?»

- ○ Aldri/Sjelden
- ○ 1
- ○ 2
- ○ 3
- ○ 4
- ○ 5
- ○ 6-7
- ○ ≥ 8

Dette elementet vises kun dersom alternativet «1», «2», «≥ 8», «3», «4», «5» eller «6-7» er valgt i spørsmålet «Hvor mange ganger pr. uke spiser du rømme, creme fraiche o.l. med lavt fettinnhold?»

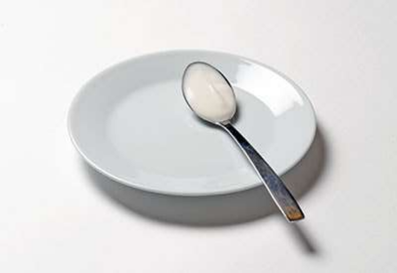

Dette elementet vises kun dersom alternativet «1», «2», «≥ 8», «3», «4», «5» eller «6-7» er valgt i spørsmålet «Hvor mange ganger pr. uke spiser du rømme, creme fraiche o.l. med lavt fettinnhold?»

Bildet over viser en spiseskje (ss) med rømme.

Hvor mye rømme, creme fraiche o.l. med lavt fettinnhold spiser du hver gang? *

Dette elementet vises kun dersom alternativet «1», «2», «≥ 8», «3», «4», «5» eller «6-7» er valgt i spørsmålet «Hvor mange ganger pr. uke spiser du rømme, creme fraiche o.l. med lavt fettinnhold?»

Oppgi mengde i spiseskjeer (ss)

- ○ 0,5 ss
- ○ 1 ss
- ○ 1 1/2 ss
- ○ 2 ss
- ○ 3 ss
- ○ ≥ 4 ss

Dette elementet vises kun dersom alternativet «Ja» er valgt i spørsmålet «Inngår yoghurt, rømme, creme fraiche eller liknende som en del av ditt ukentlige kosthold?»

Dette elementet vises kun dersom alternativet «Ja» er valgt i spørsmålet «Inngår yoghurt, rømme, creme fraiche eller liknende som en del av ditt ukentlige kosthold?»

9.2 Rømme, creme fraiche og liknende med høyt fettinnhold (f.eks. seterrømme, creme fraiche, eller inneholder mer enn 20% fett)

Hvor mange ganger pr. uke spiser du rømme, creme fraiche o.l. med høyt fettinnhold? *

Dette elementet vises kun dersom alternativet «Ja» er valgt i spørsmålet «Inngår yoghurt, rømme, creme fraiche eller liknende som en del av ditt ukentlige kosthold?»

- ○ Aldri/Sjelden
- ○ 1
- ○ 2
- ○ 3
- ○ 4
- ○ 5
- ○ 6-7
- ○ ≥ 8

Hvor mye rømme, creme fraiche o.l. med høyt fettinnhold spiser du hver gang? *

Dette elementet vises kun dersom alternativet «1», «2», «3», «4», «5», «6-7» eller «≥ 8» er valgt i spørsmålet «Hvor mange ganger pr. uke spiser du rømme, creme fraiche o.l. med høyt fettinnhold?»

Oppgi mengde i spiseskjeer (ss)

- 0,5 ss
- 1 ss
- 1 1/2 ss
- 2 ss
- 3 ss
- ≥ 4 ss

Sideskift

Obligatoriske felter er merket med stjerne *

10. Ris og pasta

Inngår ris eller pasta som en del av ditt ukentlig kosthold? *

- Ja
- Nei

Dette elementet vises kun dersom alternativet «Ja» er valgt i spørsmålet «Inngår ris eller pasta som en del av ditt ukentlig kosthold?»

10.1 Brun ris (upolert, fullkorn)

Hvor mange ganger pr. uke spiser du brun ris? *

Dette elementet vises kun dersom alternativet «Ja» er valgt i spørsmålet «Inngår ris eller pasta som en del av ditt ukentlig kosthold?»

- Aldri/Sjelden
- 1
- 2
- 3
- 4
- 5
- 6-7
- ≥ 8

Hvor mye brun ris spiser du hver gang? *

Dette elementet vises kun dersom alternativet «1», «2», «3», «4», «5», «6-7» eller «≥ 8» er valgt i spørsmålet «Hvor mange ganger pr. uke spiser du brun ris?»

Risen i bildene nedenfor er servert på en middagstallerken (19 cm).

Oppgi mengde som kokt ris.

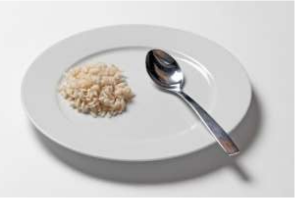

A = ca. 40 gram

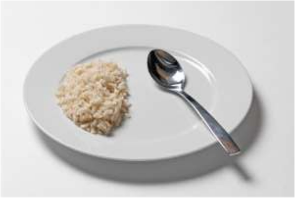

B = ca. 80 gram

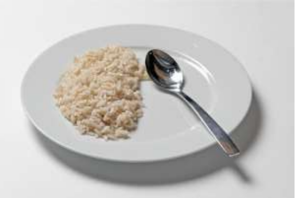

C =ca. 160 gram

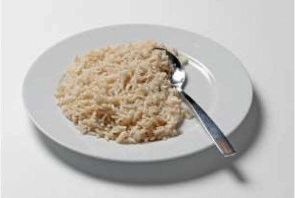

D = ca. 320 gram

Dette elementet vises kun dersom alternativet «Ja» er valgt i spørsmålet «Inngår ris eller pasta som en del av ditt ukentlig kosthold?»

Dette elementet vises kun dersom alternativet «Ja» er valgt i spørsmålet «Inngår ris eller pasta som en del av ditt ukentlig kosthold?»

10.2 Hvit ris (polert)

Hvor mange ganger pr. uke spiser du hvit ris *

Dette elementet vises kun dersom alternativet «Ja» er valgt i spørsmålet «Inngår ris eller pasta som en del av ditt ukentlig kosthold?»

- Aldri/Sjelden
- 1
- 2
- 3
- 4
- 5
- 6-7
- ≥ 8

Hvor mye hvit ris spiser du hver gang? *

Dette elementet vises kun dersom alternativet «5», «6-7», «≥ 8», «1», «2», «3» eller «4» er valgt i spørsmålet «Hvor mange ganger pr. uke spiser du hvit ris»

Risen i bildene nedenfor er servert på en middagstallerken (19 cm).

Oppgi mengde som kokt ris.

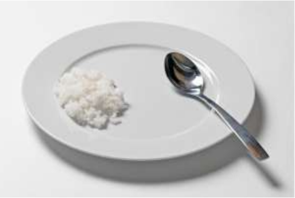

A = ca. 40 gram

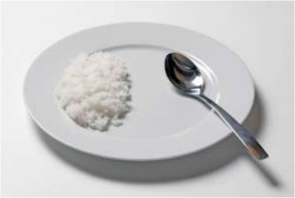

B = ca. 80 gram

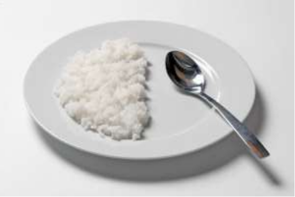

C = ca. 160 gram

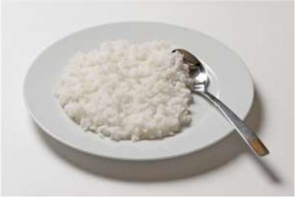

D = ca. 320 gram

Dette elementet vises kun dersom alternativet «Ja» er valgt i spørsmålet «Inngår ris eller pasta som en del av ditt ukentlig kosthold?»

Dette elementet vises kun dersom alternativet «Ja» er valgt i spørsmålet «Inngår ris eller pasta som en del av ditt ukentlig kosthold?»

10.3 Fullkornspasta

Hvor mange ganger pr. uke spiser du fullkornspasta? *

Dette elementet vises kun dersom alternativet «Ja» er valgt i spørsmålet «Inngår ris eller pasta som en del av ditt ukentlig kosthold?»

- Aldri/Sjelden
- 1
- 2
- 3
- 4
- 5
- 6-7
- ≥ 8

Hvor mye fullkornspasta spiser du hver gang? *

Dette elementet vises kun dersom alternativet «5», «6-7», «≥ 8», «1», «2», «3» eller «4» er valgt i spørsmålet «Hvor mange ganger pr. uke spiser du fullkornspasta?»

Pastaen i bildene nedenfor er servert på en middagstallerken (19 cm).

Oppgi mengde som kokt pasta.

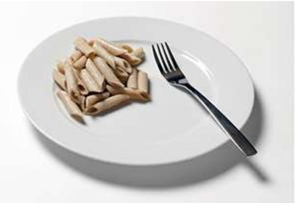

○ A = ca. 50 gram

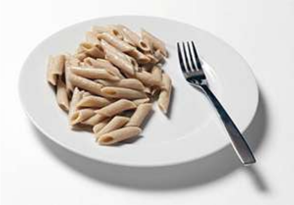

○ B = ca. 100 gram

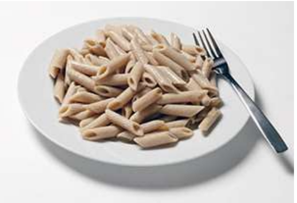

○ C = ca. 195 gram

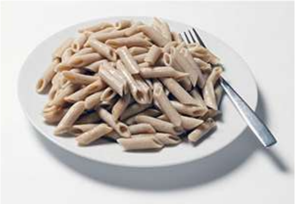

○ D = ca. 390 gram

Dette elementet vises kun dersom alternativet «Ja» er valgt i spørsmålet «Inngår ris eller pasta som en del av ditt ukentlig kosthold?»

Dette elementet vises kun dersom alternativet «Ja» er valgt i spørsmålet «Inngår ris eller pasta som en del av ditt ukentlig kosthold?»

10.4 Hvit pasta (ikke fullkorn)

Hvor mange ganger pr. uke spiser du hvit pasta? *

Dette elementet vises kun dersom alternativet «Ja» er valgt i spørsmålet «Inngår ris eller pasta som en del av ditt ukentlig kosthold?»

○ Aldri/Sjelden ○ 1 ○ 2 ○ 3

○ 4 ○ 5 ○ 6-7 ○ ≥ 8

Hvor mye hvit pasta spiser du hver gang? *

> Dette elementet vises kun dersom alternativet «5», «6-7», «≥ 8», «1», «2», «3» eller «4» er valgt i spørsmålet «Hvor mange ganger pr. uke spiser du hvit pasta?»

Pastaen i bildene nedenfor er servert på en middagstallerken (19 cm).

Oppgi mengde som kokt pasta.

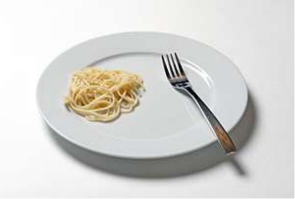

○ A = ca. 50 gram

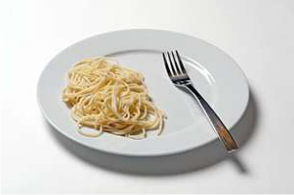

○ B = ca. 100 gram

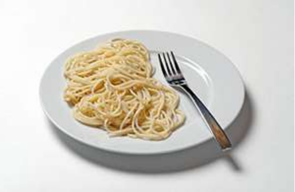

○ C = ca. 195 gram

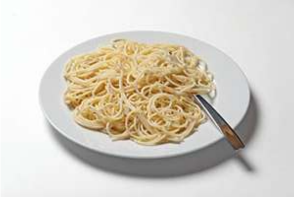

○ D = ca. 390 gram

Sideskift

Obligatoriske felter er merket med stjerne *

> Dette elementet vises kun dersom alternativet «Ja» er valgt i spørsmålet «Inngår ris eller pasta som en del av ditt ukentlig kosthold?»

---

11. Kalde og varme drikker

Vi vil først spørre deg om kalde drikker, og deretter varme drikker.

11.1 Kalde drikker i glass

Hvor ofte drikker du vanligvis de ulike typene drikker i listen under?

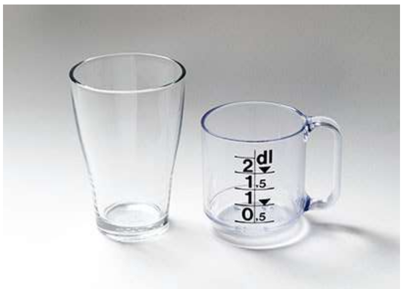

Bildet over viser et vanlig kjøkkenglass tilsvarende 2 dl.

"Glass/uke" betyr antall glass i uken, "glass/dag" betyr antall glass pr. dag. "≥7 glass/dag" betyr 7 eller flere glass pr. dag.

Husk å ta med melk du bruker på frokostgryn, grøt og dessert.

	Aldri/Sjelden	1-3 glass/uke	4-6 glass/uke	1-2 glass/dag	3-4 glass/dag	5-6 glass/dag	≥ 7 glass/dag
Vann (springvann) *	○	○	○	○	○	○	○
Flaskevann med og uten kullsyre (f.eks. Farris, Imsdal) *	○	○	○	○	○	○	○
Helmelk, kefir, kulturmelk *	○	○	○	○	○	○	○
Lettmelk (1% eller 0,5%), skummet melk, skummet kulturmelk *	○	○	○	○	○	○	○
Juice (f.eks. eplejuice, appelsinjuice uten tilsatt sukker) *	○	○	○	○	○	○	○
Saft og iste med tilsatt sukker *	○	○	○	○	○	○	○
Saft og iste uten tilsatt sukker, kunstig søtet *	○	○	○	○	○	○	○
Annen drikke uten tilsatt sukker (f.eks.lettbrus) *	○	○	○	○	○	○	○
Annen drikke med tilsatt sukker (f.eks. brus, nektar, energidrikke) *	○	○	○	○	○	○	○

11.2 Kaffe og te

Inngår kaffe eller te som en del av ditt ukentlige kosthold? *

○ Ja

○ Nei

Dette elementet vises kun dersom alternativet «Ja» er valgt i spørsmålet «Inngår kaffe eller te som en del av ditt ukentlige kosthold?»

Hvor ofte drikker du vanligvis de ulike typene drikker i listen under?

Dette elementet vises kun dersom alternativet «Ja» er valgt i spørsmålet «Inngår kaffe eller te som en del av ditt ukentlige kosthold?»

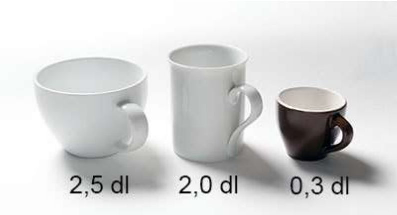

Dette elementet vises kun dersom alternativet «Ja» er valgt i spørsmålet «Inngår kaffe eller te som en del av ditt ukentlige kosthold?»

1 kopp te tilsvarer ca. 2,5 dl

1 kopp vanlig kaffe tilsvarer ca. 2,0 dl

1 kopp espresso tilsvarer ca. 0,3 dl

Dette elementet vises kun dersom alternativet «Ja» er valgt i spørsmålet «Inngår kaffe eller te som en del av ditt ukentlige kosthold?»

	Aldri/Sjelden	1-3 kopper/uke	4-6 kopper/uke	1-2 kopper/dag	3-4 kopper/dag	5-6 kopper/dag	≥ 7 kopper/dag
Kaffe (traktet,filter) *	◯	◯	◯	◯	◯	◯	◯
Presskanne kaffe, kokekaffe, kaffe-kapsel *	◯	◯	◯	◯	◯	◯	◯
Espresso *	◯	◯	◯	◯	◯	◯	◯
Annen kaffe (f.eks. cappucino, caffe latte, macchiato, andre espresso re-latert) *	◯	◯	◯	◯	◯	◯	◯
Te (f.eks. svart, grønn) *	◯	◯	◯	◯	◯	◯	◯

Sideskift

Obligatoriske felter er merket med stjerne *

12. Alkoholholdige drikker

Drikker du vanligvis alkoholholdige drikker? *

- ◯ Ja
- ◯ Nei, vanligvis ikke

Dette elementet vises kun dersom alternativet «Ja» er valgt i spørsmålet «Drikker du vanligvis alkoholholdige drikker?»

Hvor mange ganger pr. uke drikker du alkoholholdige drikker?

Dette elementet vises kun dersom alternativet «Ja» er valgt i spørsmålet «Drikker du vanligvis alkoholholdige drikker?»

"gang/uke" betyr antall ganger i uken.

Dette elementet vises kun dersom alternativet «Ja» er valgt i spørsmålet «Drikker du vanligvis alkoholholdige drikker?»

	Aldri/Sjelden	1 gang/uke	2 ganger/uke	3 ganger/uke	4 ganger/uke	5 ganger/uke	6-7 ganger/uke	≥ 8 gang/uke
Øl, sterk øl, pils *	◯	◯	◯	◯	◯	◯	◯	◯
Vin *	◯	◯	◯	◯	◯	◯	◯	◯
Brennevin *	◯	◯	◯	◯	◯	◯	◯	◯

Dette elementet vises kun dersom alternativet «Ja» er valgt i spørsmålet «Drikker du vanligvis alkoholholdige drikker?»

Hvor mye drikker du hver gang?

Dette elementet vises kun dersom alternativet «Ja» er valgt i spørsmålet «Drikker du vanligvis alkoholholdige drikker?»

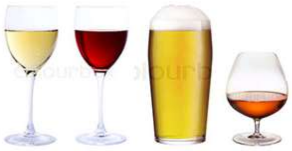

Dette elementet vises kun dersom alternativet «Ja» er valgt i spørsmålet «Drikker du vanligvis alkoholholdige drikker?»

Bildet over viser vanlig vinglass tilsvarende 1,2 dl, ølglass tilsvarende 4,0 dl og brennevinsglass tilsvarende 0,4 dl.

Oppgi mengde i antall glass.

Dette elementet vises kun dersom alternativet «Ja» er valgt i spørsmålet «Drikker du vanligvis alkoholholdige drikker?»

	0	1/2 glass	1 glass	2 glass	3 glass	4 glass	5 glass	≥ 6 glass
Øl, sterk øl, pils (glass, 4 dl) *	○	○	○	○	○	○	○	○
Vin (glass, 1,2 dl) *	○	○	○	○	○	○	○	○
Brennevin (glass, 0,4 dl)	○	○	○	○	○	○	○	○

Sideskift

Obligatoriske felter er merket med stjerne *

13. Kaker, dessert, godteri

Inngår kaker, dessert og godteri i ditt ukentlige kosthold? *

- ○ Ja
- ○ Nei

Dette elementet vises kun dersom alternativet «Ja» er valgt i spørsmålet «Inngår kaker, dessert og godteri i ditt ukentlige kosthold?»

Hvor ofte spiser du vanligvis de ulike matvarene i listen under?

Dette elementet vises kun dersom alternativet «Ja» er valgt i spørsmålet «Inngår kaker, dessert og godteri i ditt ukentlige kosthold?»

	Aldri/Sjelden	1 enhet/uke	2-3 enheter/uke	4-5 enheter/uke	6-7 enheter/uke	8-9 enheter/uke	≥ 10 enheter/uke
Kaker, hvetebakst, vafler, søt kjeks (1 enhet = ca. 80 gram, 1 kakestykke= 1 bolle= 1 vaffelplate= 8 små kjeks) *	○	○	○	○	○	○	○
Dessert (f.eks. is, hermetisk frukt, pudding) (1 enhet= 1,2 dl) *	○	○	○	○	○	○	○
Sjokolade, godteri (1 porsjon= 100gram) *	○	○	○	○	○	○	○
Potetull, chips (1 enhet= 1 neve= 15 gram) *	○	○	○	○	○	○	○

Sideskift

Obligatoriske felter er merket med stjerne *

14. Kosttilskudd

Inngår kosttilskudd i ditt ukentlige kosthold? *

- ○ Ja
- ○ Nei

Dette elementet vises kun dersom alternativet «Ja» er valgt i spørsmålet «Inngår kosttilskudd i ditt ukentlige kosthold?»

Hvor ofte spiser du kosttilskuddene i listen under?

Dette elementet vises kun dersom alternativet «Ja» er valgt i spørsmålet «Inngår kosttilskudd i ditt ukentlige kosthold?»

	Aldri/Sjelden	1-2 enheter/uke	3-4 enheter/uke	1 enhet/dag	2 enheter/dag	3 enheter/dag	≥ 4 enheter/dag
Tran (1 enhet= 1 barneeskje) *	○	○	○	○	○	○	○
Trankapsler, fiskeoljekapsler, omega-3 tilskudd (1 enhet=1 kap-sel) *	○	○	○	○	○	○	○
Vitamin D (1 enhet= 1 pille) *	○	○	○	○	○	○	○
Mulitvitamin tilskudd (1 enhet= 1 pille) *	○	○	○	○	○	○	○
Jern (1 enhet= 1 pille) *	○	○	○	○	○	○	○
Kalsium (1 enhet= 1 pille) *	○	○	○	○	○	○	○
Andre kosttilskudd *	○	○	○	○	○	○	○

Sideskift

Obligatoriske felter er merket med stjerne *

15. Fysisk aktivitet, tid i ro og søvn

Vi vil først spørre deg om din fysiske aktivitet der du blir lett og veldig andpusten, og deretter hvor lenge du er i ro og sover i løpet av ett vanlig døgn.

15.1 Hvor mange ganger pr. uke er du fysisk aktiv der du blir lett andpusten (moderat intensitet)? *

F.eks. hurtig gange, hardt husarbeid, fysisk aktiv i arbeid

- ○ Aldri/Sjelden
- ○ 1
- ○ 2
- ○ 3
- ○ 4
- ○ 5
- ○ 6-7
- ○ ≥ 8

15.1.1 Hvor lenge var du fysisk aktiv hver gang (minutter) i moderat intensitet? *

Dette elementet vises kun dersom alternativet «4», «5», «6-7», «≥ 8», «1», «2» eller «3» er valgt i spørsmålet «15.1 Hvor mange ganger pr. uke er du fysisk aktiv der du blir lett andpusten (moderat intensitet)?»

- ○ 1-4 minutter
- ○ 5-9 minutter
- ○ 10-15 minutter
- ○ 16-20 minutter
- ○ 21-30 minutter
- ○ 31-45 minutter
- ○ 46-60 minutter
- ○ ≥ 61 minutter

15.2 Hvor mange ganger pr. uke er du fysisk aktiv der du blir veldig andpusten (høy intensitet)? *

F.eks. jogging, skigåing, hard fysisk aktivitet, driver idrett

- ○ Aldri/Sjelden
- ○ 1
- ○ 2
- ○ 3
- ○ 4
- ○ 5
- ○ 6-7
- ○ ≥ 8

15.2.2 Hvor lenge var du fysisk aktiv hver gang (minutter) i høy intensitet? *

Dette elementet vises kun dersom alternativet «4», «5», «6-7», «≥ 8», «1», «2» eller «3» er valgt i spørsmålet «15.2 Hvor mange ganger pr. uke er du fysisk aktiv der du blir veldig andpusten (høy intensitet)?»

- ○ 1-4 minutter
- ○ 5-9 minutter
- ○ 10-15 minutter
- ○ 16-20 minutter
- ○ 21-30 minutter
- ○ 31-45 minutter
- ○ 46-60 minutter
- ○ ≥ 61 minutter

Vi vil nå spørre deg om hvor lenge du vanligvis er i ro og sover i løpet av ett døgn (24 timer).

Summen av de tre neste spørsmålene kan ikke bli mer enn 24 timer som tilsvarer ett døgn.

15.3 Hvor mange timer sitter du i ro i løpet av en vanlig arbeidsdag? *

Med ro menes stillesittende aktivitet, f.eks. transport til og fra arbeid (bil, tog, buss, trikk etc.), å lese dokumenter, tid brukt til måltider, sitter i møter, sitter foran PC, sitter med en mobiltelefon eller en annen skjerm.

Hvis du ikke er i arbeid kan du svare 0 (null) her og gå til neste spørsmål.

15.4 Hvor mange timer sitter du i ro i løpet av din fritid eller i løpet av en vanlig dag? *

Med ro menes stillesittende aktivitet, f.eks. å lese bok, tid brukt til måltider, sitter eller ligger og ser på TV, sitter med en PC, mobiltelefon eller annen skjerm.

15.5 Hvor mange timer sover du vanligvis pr. døgn? *

Sideskift

Obligatoriske felter er merket med stjerne *

16. Røykevaner

Hva passer best for å beskrive dine røykevaner nå? *

- ◯ Røyker daglig
- ◯ Røyker av og til
- ◯ Har sluttet helt å røyke
- ◯ Har aldri røykt verken daglig eller av og til

Hvor mange år er det siden du sluttet å røyke siste gang? *

Dette elementet vises kun dersom alternativet «Har sluttet helt å røyke» er valgt i spørsmålet «Hva passer best for å beskrive dine røykevaner nå?»

Hvor mange år har du røykt sammenhengende? Trekk fra de periodene du ikke har røykt, hvis du har sluttet å røyke i lengre perioder *

Dette elementet vises kun dersom alternativet «Røyker daglig», «Røyker av og til» eller «Har sluttet helt å røyke» er valgt i spørsmålet «Hva passer best for å beskrive dine røykevaner nå?»

- ◯ 1-5 år
- ◯ 6-10 år
- ◯ 11-15 år
- ◯ 16-20 år
- ◯ 21-25 år
- ◯ Mer enn 25 år

Antall sigaretter *

Dette elementet vises kun dersom alternativet «Røyker daglig» er valgt i spørsmålet «Hva passer best for å beskrive dine røykevaner nå?»

Hvor mange sigaretter røyker du i gjennomsnitt pr. dag?

Antall sigaretter *

Dette elementet vises kun dersom alternativet «Røyker av og til» er valgt i spørsmålet «Hva passer best for å beskrive dine røykevaner nå?»

Hvor mange sigaretter røyker du anslagsvis pr. uke?

Antall sigaretter *

Dette elementet vises kun dersom alternativet «Har sluttet helt å røyke» er valgt i spørsmålet «Hva passer best for å beskrive dine røykevaner nå?»

Hvor mange sigaretter pleide du å røyke pr. uke før du sluttet?

Sideskift

Obligatoriske felter er merket med stjerne *

17. Snus

Hva passer best for å beskrive dine snusvaner: *

- Bruker snus daglig
- Bruker snus av og til
- Har sluttet å bruke snus
- Har aldri brukt snus

Hvor mange år er det siden du sluttet å snuse siste gang? *

Dette elementet vises kun dersom alternativet «Har sluttet å bruke snus» er valgt i spørsmålet «Hva passer best for å beskrive dine snusvaner:»

Hvor mange år har du snuset sammenhengende, trekk fra de periodene du ikke har snust, hvis du har sluttet å snuse i lengre perioder. *

Dette elementet vises kun dersom alternativet «Bruker snus daglig», «Bruker snus av og til» eller «Har sluttet å bruke snus» er valgt i spørsmålet «Hva passer best for å beskrive dine snusvaner:»

- 1-5 år
- 6-10 år
- 11-15 år
- 16-20 år
- 21-25 år
- Mer enn 25 år

Hvor mange bokser med snus bruker du pr. dag? *

Dette elementet vises kun dersom alternativet «Bruker snus daglig» er valgt i spørsmålet «Hva passer best for å beskrive dine snusvaner:»

Hvor mange bokser med snus bruker du pr. uke? *

Dette elementet vises kun dersom alternativet «Bruker snus av og til» er valgt i spørsmålet «Hva passer best for å beskrive dine snusvaner:»

Hvor mange bokser med snus brukte du pr. uke før du sluttet? *

Dette elementet vises kun dersom alternativet «Har sluttet å bruke snus» er valgt i spørsmålet «Hva passer best for å beskrive dine snusvaner:»

Sideskift

Obligatoriske felter er merket med stjerne *

18. Generelle opplysninger

Alder *

Vennligst oppgi alder i år.

Kjønn *

- Mann
- Kvinne

Vekt *

Vennligst oppgi vekt i kg.

Høyde *

Vennligst oppgi høyde i cm.

Bosituasjon *

- Bor sammen med en eller flere
- Bor alene

Hvilken utdanning er den høyeste du har fullført? *

- Grunnskole 7-10 år, framhaldsskole, folkehøgskole
- Realskole, middelskole, yrkesskole, 1-2 årig videregående
- Artium, økonomisk gymnas, allmennfaglig retning
- Høgskole/Universitet, mindre enn 4 år
- Høgskole/Universitet, 4 år eller mer
- Fagbrev

Arbeidsførhet *

- ◯ I arbeid (helt eller delvis)
- ◯ Hjemmeværende (selvvalgt)
- ◯ Pensjonist
- ◯ Arbeidsledig
- ◯ Sykmeldt
- ◯ Under attføring/rehabilitering
- ◯ Midlertidig uføretrygdet
- ◯ Varig uføretrygdet
- ◯ Student

Hva slags etnisk bakgrunn har din far? *

Flere svar er mulig, hvis annet spesifiser i neste spørsmål

- ◯ Europa
- ◯ Afrika
- ◯ Asia
- ◯ Annet

Hvis annen etnisk bakgrunn, spesifiser her:

i Dette elementet vises kun dersom alternativet «Annet» er valgt i spørsmålet «Hva slags etnisk bakgrunn har din far?»

Hva slags etnisk bakgrunn har din mor? *

Flere svar er mulig, hvis annet spesifiser i neste spørsmål

- ◯ Europa
- ◯ Afrika
- ◯ Asia
- ◯ Annet

Hvis annen etnisk bakgrunn, spesifiser her:

i Dette elementet vises kun dersom alternativet «Annet» er valgt i spørsmålet «Hva slags etnisk bakgrunn har din mor?»

Hvor lenge (år) har du bodd i Norge? *

Her har du mulighet til å skrive ned andre matvarer du spiser som vi ikke har spurt deg om. Dette er valgfritt.

Tusen takk for at du tok deg tid til å svare på dette spørreskjemaet!

Se nylige endringer i Nettskjema

Table S6: Technical functions in the DIGIKOST-FFQ and the DIGIKOST report

Technical functions		DIGIKOST-FFQ	DIGIKOST report
1.	Automatic digital functions promoting efficient completion time:<br>- introductive question of regular intake/use of food or activity with binary outcome (yes/no)<br>- no questions on amounts appears when ticked off zero or not using in frequency questions.	X	
2.	Automatic function enabling mandatory completeness of all questions before able to go to the next question.	X	
3.	Algorithms counting slices of bread reported in the DIGIKOST-FFQ in real time (front-end function) and immediately available for the respondents helping to report actual amounts of different spreads on the slices of bread.	X	
4.	Variables from the code-book in the DIGIKOST-FFQ automatically delivered to the secured server, TSD, as CSV file and MS Excel file. Complex algorithms, coded in R-scripts, transforming and generating variables to the DIGIKOST database (back-end function) inside the secure server, and can be exported as CSV file and a MS Excel file.	X	
5.	Complex algorithms, coded in R-scripts, generating DIGIKOST report with benchmarking to Norwegian FBDG (back-end function) inside the secure server. Available for researchers and automatically exported and sent to the participants.		X
6.	High quality images:<br>- illustrating portion sizes of different foods<br>- different food groups in DIGIKOST report<br>- lifestyle activities in DIGIKOST report	X	X
7.	Informal text to increase the understanding of the questions and individual advices on how to achieve the recommendations	X	X

Figure S3: Example of DIGIKOST report

DIGIKOST

Screening of dietary intake and other lifestyle factors

Report about your dietary intake, physical activity and other lifestyle factors

Print the report

Contents

Thank you for completing the DIGIKOST questionnaire.
Here, we present an overview of your dietary intake and physical activity and other lifestyle factors. In addition, your adherence to the Norwegian Food Based Dietary Guidelines is also presented along with advices to achieve the different recommendations.

Dietary intake and physical activity

In the table below you find your dietary intake and physical activity estimated from your answers in the DIGIKOSTquestionnaire and compared to the dietary guidelines. You will find more detailed information regarding your intake and other lifestyle factors further down in this report.

Table of dietary intake and physical activity

	Your intake	Recommended intake	Your achievement
Fruit	174 g/d	At least 250 g/d	76 grams below recommended
Vegetables	157 g/d	At least 250 g/d	93 grams below recommended
Whole grains	166 g/d	At least 90 g/d	Fulfilling recommendations
Fish	1116 g/week	At least 300 g/week	Fulfilling recommendation
Red meat	1129 g/week	Max 500 g/week	629 grams above recommended
Sugar- and fat rich foods	0 g/d	Max 20 g/d	Fulfilling recommendation
Physical activity	46 minutes/week	150 minutes/week	104 minutes below recommended

Individual advices for you

Based on your reported dietary intake and other lifestyle factors, we have made some specific advices in how to fulfil the recommendations. You can by only small adjustments increase your benefits in health.

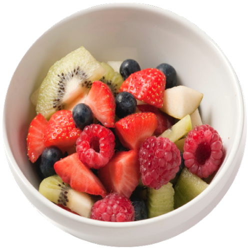

You partially follow the recommendation for fruit intake.

Your fruit intake is 174 grams per day. It is great that you eat some fruit, however, if you increase your intake with for instance one banana and a plum per day you will reach the recommended daily intake.

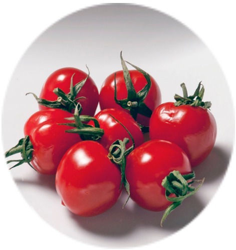

You partially follow the recommendation for vegetable intake.

Your vegetable intake is 157 grams per day.It is great that you eat some vegetables, however, if you increase your intake with for instance one carrot and a large tomato per day you will reach the recommended daily intake.

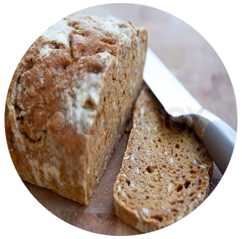

You follow the recommendation for whole grains intake.

Your whole grains intake is 166 grams per day. Keep on, this is beneficial for your health.

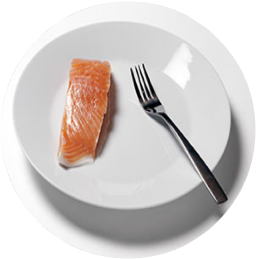

You follow the recommendation for fish intake.

Your whole grains intake is 1116 grams per week. Keep on, this is beneficial for your health.

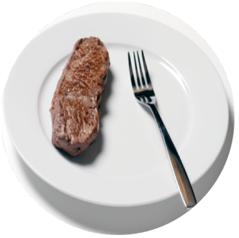

You do not follow the recommendation for red meat intake.

Your red meat intake is 1129 grams per week. If you limit your intake to eat red meat for dinner no more than 2-3 times a week, you will fulfill the recommendation.

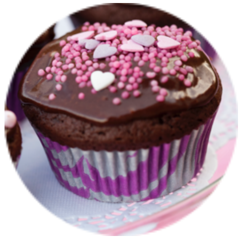

You follow the recommendation for low intakes of sugar- and fat rich foods.

Your sugar- and fat rich food intake is 0 grams per day. It is recommended to limit intakes of sugar- and fat rich foods.

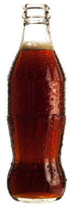

You do not follow the recommendation for low intakes of drinks with added sugar.

Your drinks with added sugar intake is 143 grams per day, and more than recommended. We advice you to limit your intakes of drinks with added sugar and replace it with drinks without added sugar to fulfill the recommendation.

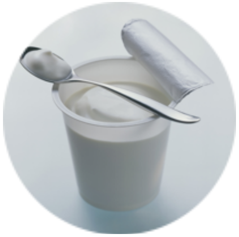

You follow the recommendation for low-fat dairy product intake.

Your low-fat dairy product intake is 165 grams per day. Keep on, this is beneficial for your health.

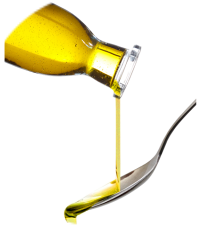

You do not follow the recommendation for using most margarine and oils.

It is recommended to have an intake of margarines and oils. If you have intake of butter, we advice you to replace this with margarine or oils in order to achieve the recommendation.

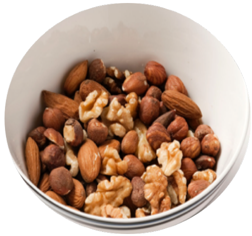

You do not follow the recommendation for unsalted nut intake.

Your unsalted nut intake is 0 grams per day. If you increase your intake with a handful of unsalted nuts per day you will reach the recommended daily intake.

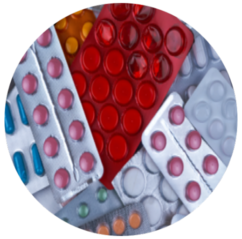

Please notice if you are adviced by your physician to use dietary supplements, you should continue to follow this recommendation.

You do not follow the recommendation for no intake of dietary supplements. By replacing dietary supplements with fresh vegetables, fruits, low-fat dairy products, whole grains, fish and lean meat you will reach the recommended daily intake of nutrients.

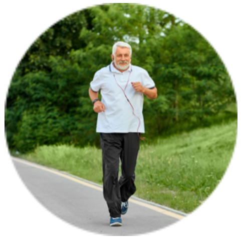

You do not follow the recommendation of being physical active.

You are 46 minutes in physical activity per week. It is recommended to be physical active in at least 150 minutes per week. We advice you to use the stairs instead of elevator and perform a brisk walk each day of at least 20 minutes in order to reach the recommendation of physical activity.

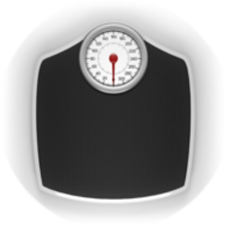

Your body weight is within the normal range.

A body weight within the normal range reduce the risk of developing chronic diseases.

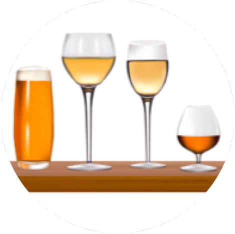

You do not follow the recommendation for alcohol intake.

Your alcohol intake is above 30 grams per week. We advice you to replace alcoholic drinks with non-alcoholic drinks in order to reach the recommendation.

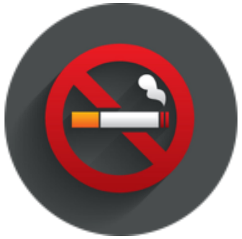

You do not follow the recommendation of not using tobacco.

The health authority give advices and information on how to stop using tobacco (http://www.helsenorge.no)

Illustrasjonsbildene er hentet fra Colourbox.no og fotograf Gunnar F. Lothe

References

32. Henriksen HB, Berg HB, Frost Andersen L, Weedon-Fekjær H, Blomhoff R. Development of the Norwegian diet index and the Norwegian lifestyle index and evaluation in a national survey. Food Nutr Res. 2023;67. doi: 10.29219/fnr.v67.9217.
31. Henriksen HB, Berntsen S, Paur I, Zucknick M, Skjetne AJ, Bøhn SK, et al. Validation of two short questionnaires assessing physical activity in colorectal cancer patients. BMC Sports Sci Med Rehabil. 2018;10:8. Epub 2018/06/02. doi: 10.1186/s13102-018-0096-2. PubMed PMID: 29854408; [Medline: 29854408].
34. Dalane JØ, Bergvatn TAM, Kielland E, Carlsen MH. Mål, vekt og porsjonsstørrelser for matvarer = Weights, measures and portion sizes for foods. Oslo: Mattilsynet Universitetet i Oslo Helsedirektoratet; 2015.
57. University Center for Information Technology (USIT) 2021. Available from: https://www.usit.uio.no/english/.
33. Nettskjema 2023 [20.01.2023]. Available from: https://nettskjema.no/.
29. Norwegian Digitalisation Agency 2023 [21.01.2023]. Available from: https://www.digdir.no/digdir/about-norwegian-digitalisation-agency/887.
26. Henriksen HB, Carlsen MH, Paur I, Berntsen S, Bohn SK, Skjetne AJ, et al. Relative validity of a short food frequency questionnaire assessing adherence to the Norwegian dietary guidelines among colorectal cancer patients. Food Nutr Res. 2018;62. Epub 2018/03/17. doi: 10.29219/fnr.v62.1306. PubMed PMID: 29545734; PubMed Central PMCID: PMCPMC5846207.
30. Difi 2023 [21.01.2023]. Available from: https://eid.difi.no/nb/id-porten.
27. Henriksen HB, Knudsen MD, Carlsen MH, Hjartåker A, Blomhoff R. A Short Digital Food Frequency Questionnaire (DIGIKOST-FFQ) Assessing Dietary Intake and Other Lifestyle Factors Among Norwegians: Qualitative Evaluation With Focus Group Interviews and Usability Testing. JMIR Formative Research. 2022. doi: 10.2196/35933.
35. Norwegian Directorate of Health [cited 2021]. Available from: https://www.helsenorge.no/kosthold-og-ernaring/sma-grep-for-et-sunt-kosthold/dagens-maltider/#tallerkenmodellen.
36. Norwegian Institute of Public Health [2021]. Available from: https://www.fhi.no/en/.
3. Kostråd for å fremme folkehelsen og forebygge kroniske sykdommer: metodologi og vitenskapelig kunnskapsgrunnlag. Oslo: Nasjonalt råd for ernæring, Helsedirektoratet; 2011. 353 s. : ill. p.
37. World Health Organization (WHO), Europe. Available from: https://www.euro.who.int/en/health-topics/disease-prevention/nutrition/a-healthy-lifestyle/body-mass-index-bmi?source=post_page.
38. Web-form, University of Oslo [2021]. Available from: https://www.uio.no/english/services/it/adm-services/nettskjema/.
