# Supplementary material for: Digital Food Frequency Questionnaire Assessing Adherence to the Norwegian Food–Based Dietary Guidelines and Other National Lifestyle Recommendations: Instrument Validation Study
Source: J Med Internet Res. 2024 Apr 30;26:e53442. doi: 10.2196/53442 (PMC11094607; doi:10.2196/53442)
Supplement: Multimedia Appendix 2 [file jmir_v26i1e53442_app2.docx]

| **Variables** | **DIGIKOST-FFQ,**  **mean (±SD) (min, max)** | **WR,**  **mean (±SD) (min, max)** |
| --- | --- | --- |
| Diet score (total)^1^ | 11.2 (3.2) (4,18) | 10.4 (3.1)( (4.5, 20) |
| Fruits and berries^2^ | 1.7 (1.2) (0,3) | 1.8 (1.3) (0,3) |
| Vegetables^2^ | 1.9 (1.1) (0,3) | 1.1 (1.1) (0,3) |
| Unsalted nuts^2^ | 0.8 (1.2) (0,3) | 0.4 (0.8) (0,3) |
| Whole grains^2^ | 2.3 (1.0) (0,3) | 1.1 (1.1) (0,3) |
| Fish (lean and fatty)^3^ | 0.8 (0.4) (0,1) | 0.6 (0.4) (0,1) |
| Red meat (processed and unprocessed)^3^ | 0.7 (0.4) (0,1) | 0.5 (0.4) (0,1) |
| Processed meat (white and red)^3^ | 0.3 (0.4) (0,1) | 0.3 (0.4) (0,1) |
| Sugar- and fat rich foods^3^ | 0.3 (0.4) (0,1) | 0.1 (0.2) (0,1) |
| Drinks with added sugar^3^ | 0.8 (0.4) (0,1) | 0.6 (0.5) (0,1) |
| Low fat dairy products^3^ | 0.5 (0.4) (0,1) | 0.7 (0.4) (0,1) |
| Margarines, butter and oils^3^ | 0.7 (0.4) (0,1) | 0.6 (0.4) (0,1) |
| Dietary supplements^3^ | 0.5 (0.5) (0,1) | 0.8 (0.4) (0,1) |

FFQ: food frequency questionnaire, WR: weighed record

^1^score ranging from 0-20

^2^scores ranging from 0-3

^3^scores ranging from 0-1
